# First-in-human study of oleclumab, a potent, selective anti-CD73 monoclonal antibody, alone or in combination with durvalumab in patients with advanced solid tumors

**DOI:** 10.1007/s00262-023-03430-6

**Published:** 2023-04-05

**Authors:** Johanna Bendell, Patricia LoRusso, Michael Overman, Anne M. Noonan, Dong-Wan Kim, John H. Strickler, Sang-We Kim, Stephen Clarke, Thomas J. George, Peter S. Grimison, Minal Barve, Manik Amin, Jayesh Desai, Trisha Wise-Draper, Steven Eck, Yu Jiang, Anis A. Khan, Yuling Wu, Philip Martin, Zachary A. Cooper, Nairouz Elgeioushi, Nancy Mueller, Rakesh Kumar, Sandip Pravin Patel

**Affiliations:** 1 Sarah Cannon Research Institute, Nashville, TN USA; 2 Yale University Cancer Center, New Haven, CT USA; 3 University of Texas MD Anderson Cancer Center, Houston, TX USA; 4 Ohio State University, Wexner Medical Center, James Comprehensive Cancer Center, Columbus, OH USA; 5 Seoul National University Hospital, Seoul, South Korea; 6 Duke University Medical Center, Durham, NC USA; 7 Asan Medical Center, Seoul, South Korea; 8 Royal North Shore Hospital, St. Leonards, NSW Australia; 9 University of Florida Health Cancer Center, Gainesville, FL USA; 10 Chris O’Brien Lifehouse, Camperdown, NSW Australia; 11 Mary Crowley Cancer Research, Dallas, TX USA; 12 Washington University School of Medicine, St. Louis, MO USA; 13 Royal Melbourne Hospital, Parkville, VIC Australia; 14 University of Cincinnati Cancer Center, Cincinnati, OH USA; 15 AstraZeneca, Gaithersburg, MD USA; 16 Moores Cancer Center, University of California San Diego, La Jolla, San Diego, CA USA; 17 Roche Innovation Center Basel, F. Hoffmann-La Roche Ltd, Basel, Switzerland; 18 Dartmouth-Hitchcock Medical Center, Norris Cotton Cancer Center, Lebanon, NH USA

**Keywords:** Adenosine, CD73, Monoclonal antibody, Oleclumab, Tumor microenvironment

## Abstract

**Background:**

CD73 upregulation in tumors leads to local immunosuppression. This phase I, first-in-human study evaluated oleclumab (MEDI9447), an anti-CD73 human IgG1λ monoclonal antibody, alone or with durvalumab in patients with advanced colorectal cancer (CRC), pancreatic ductal adenocarcinoma (PDAC), or epidermal growth factor receptor-mutant non-small-cell lung cancer (NSCLC).

**Methods:**

Patients received oleclumab 5–40 mg/kg (dose-escalation) or 40 mg/kg (dose-expansion) intravenously every 2 weeks (Q2W), alone (escalation only) or with durvalumab 10 mg/kg intravenously Q2W.

**Results:**

192 patients were enrolled, 66 during escalation and 126 (42 CRC, 42 PDAC, 42 NSCLC) during expansion. No dose-limiting toxicities occurred during escalation. In the monotherapy and combination therapy escalation cohorts, treatment-related adverse events (TRAEs) occurred in 55 and 54%, respectively, the most common being fatigue (17 and 25%). In the CRC, PDAC, and NSCLC expansion cohorts, 60, 57, and 45% of patients had TRAEs, respectively; the most common were fatigue (15%), diarrhea (9%), and rash (7%). Free soluble CD73 and CD73 expression on peripheral T cells and tumor cells showed sustained decreases, accompanied by reduced CD73 enzymatic activity in tumor cells. Objective response rate during escalation was 0%. Response rates in the CRC, PDAC, and NSCLC expansion cohorts were 2.4% (1 complete response [CR]), 4.8% (1 CR, 1 partial response [PR]), and 9.5% (4 PRs), respectively; 6-month progression-free survival rates were 5.4, 13.2, and 16.0%.

**Conclusions:**

Oleclumab ± durvalumab had a manageable safety profile, with pharmacodynamic activity reflecting oleclumab’s mechanism of action. Evidence of antitumor activity was observed in tumor types that are generally immunotherapy resistant.

**Clinical trial registration:**

Clinicaltrials.gov, NCT02503774; date of registration, July 17, 2015.

**Supplementary Information:**

The online version contains supplementary material available at 10.1007/s00262-023-03430-6.

## Introduction

The enzyme cluster of differentiation 73 (CD73) is an important component of the regulatory signaling network mediating immune activation and suppression in the cellular microenvironment [1–3]. CD73 hydrolyzes adenosine monophosphate to adenosine, which is involved in promotion of cell growth and immunosuppression via purinergic G protein coupled adenosine receptors [1, 2]. Multiple tumor types express high levels of CD73 and adopt this system for evasion of immune surveillance [3, 4], including colorectal cancer (CRC) [5], pancreatic ductal adenocarcinoma (PDAC) [5, 6], and lung adenocarcinoma [7], notably epidermal growth factor receptor-mutant (EGFRm) adenocarcinoma [8, 9], with upregulation of CD73 increasing extracellular adenosine production resulting in local immunosuppression [3, 10]. Furthermore, elevated tumor CD73 expression is associated with worse outcomes in several cancers [1, 11], including PDAC [6, 12] and CRC [13, 14], and CD73 may be among the drivers of tumor metastasis [4, 15].

Immunotherapy is transforming cancer treatment, but durable response is seen in a limited proportion of patients as primary or acquired resistance to treatment represents a major challenge [16]. Understanding the molecular mechanisms behind such resistance may enable rational combinatorial approaches that (re-)sensitize tumors to specific agents [5]. Targeting of CD73 offers potential in this context [17–19]; as CD73 protein expression may be increased in treatment-resistant cancer cells and host-derived cells such as immunosuppressive Tregs in the tumor microenvironment, CD73 blockade may result in immune stimulation and antitumor activity [4, 10]. Preclinical studies support this hypothesis [18]: CD73 blockade significantly enhances the activity of anti-programmed cell death (PD)-1 and anti-cytotoxic T-lymphocyte antigen-4 monoclonal antibodies in syngeneic mouse models of colon, prostate, and breast cancers [20], an anti-CD73 monoclonal antibody inhibits tumor growth and reverses the exhausted T-cell phenotype in an immunocompetent transgenic head and neck squamous cell carcinoma mouse model [21], and anti-CD73 antibody treatment has been shown to enhance gemcitabine efficacy in orthotopic PDAC mouse models [22].

Oleclumab (MEDI9447) is a human IgG1λ monoclonal antibody that potently and selectively inhibits the catalytic activity of CD73 via steric blocking and inter-CD73 dimer crosslinking, and decreases CD73 expression through internalization, thereby inhibiting the production of immunosuppressive extracellular adenosine [23–25]. Oleclumab as monotherapy and in combination with anti-PD-1/PD-ligand (L)-1 antibodies and chemotherapy has been shown to inhibit tumor growth across a variety of models through increased anti-tumor immune activation [9, 25, 26]. These findings provide the rationale for this first-in-human study of oleclumab alone or in combination with the anti-PD-L1 monoclonal antibody durvalumab in patients with advanced CRC, PDAC, or EGFRm non-small-cell lung cancer (NSCLC) given the high expression of CD73 and its association with poor prognosis in these tumor types.

## Methods

### Study design

This was a phase I, multicenter, open-label, dose-escalation and dose-expansion study (NCT02503774). The primary objectives were to assess safety and tolerability, describe any dose-limiting toxicity (DLT), and determine the maximum tolerated dose (MTD) of oleclumab alone and with durvalumab. Key secondary objectives were to determine the pharmacokinetics (PK) of oleclumab alone and with durvalumab; to determine immunogenicity in terms of the percentage of patients developing antidrug antibodies (ADAs); to evaluate candidate pharmacodynamic biomarkers in tumor tissue via assessment of target expression; and to describe preliminary antitumor activity in terms of objective response (OR) and disease control (DC) rates based on Response Evaluation Criteria in Solid Tumors (RECIST) v1.1 [27], duration of response (DoR), progression-free survival (PFS), and overall survival (OS). Exploratory objectives included evaluation of additional candidate biomarkers such as soluble CD73 in serum, and their relationships with clinical outcomes.

### Patients

Patients were aged ≥ 18 years and had histologically/cytologically confirmed CRC, PDAC, or NSCLC; ≥ 1 measurable lesion per RECIST v1.1 [27]; an Eastern Cooperative Oncology Group performance score of ≤ 1; and adequate hematologic, renal, and hepatic function (Supplementary Methods). Patients with CRC or PDAC were required to have progressed on, be refractory to, or intolerant of standard-of-care therapy and, in the escalation phase, to have received ≤ 5 prior lines of therapy. Additional criteria in the expansion phase were: (1) patients with CRC or PDAC required positive CD73 expression by immunohistochemistry (using a validated assay; Supplementary Methods) on ≥ 10% of tumor cells (a requirement reviewed at each interim analysis); (2) CRC tumors were required to be microsatellite-stable (MSS) or mismatch repair-proficient and NSCLC tumors were required to harbor EGFR mutations; and (3) patients with CRC must have received 2–4 prior lines, patients with PDAC 1–2 prior lines, and patients with EGFRm NSCLC 1–4 prior lines of systemic therapy in the metastatic setting (Supplementary Methods).

All patients provided written, informed consent to participate in the study, including consent to provide archived tumor specimens from core biopsy or larger resection < 3 years from screening for biomarker studies. All patients were requested to provide optional consent for paired pretreatment and on-treatment tumor biopsies where clinically feasible (as determined by the treating physician). The study protocol was approved by the institutional review board or ethics committee for each participating center, and the study was run in accordance with ethical principles originating in the Declaration of Helsinki and consistent with the International Conference on Harmonization guidelines on Good Clinical Practice, as well as applicable regulatory requirements.

### Treatment

Dose escalation proceeded using a 3 + 3 design. Patients received oleclumab 5, 10, 20, or 40 mg/kg via intravenous infusion every 2 weeks (Q2W), either as monotherapy or in combination with durvalumab 10 mg/kg intravenously Q2W (≥ 15 min after end of oleclumab infusion), until disease progression, unacceptable toxicity, death, or patient withdrawal. Dose escalation in the combination therapy cohort commenced following completion of the DLT evaluation period at the third dose level in the monotherapy cohort (Supplementary Methods for DLT definition). In the expansion phase, patients received combination therapy on the same schedule and at oleclumab 40 mg/kg.

### Assessments

Safety was monitored regularly from the signing of the informed consent form until 30 days post end of treatment (and then at 12 weeks post end of treatment) by physical examination, electrocardiogram, assessment of blood hematology, clinical chemistry, urinalysis, vital signs, and assessment of adverse events (AEs)/serious AEs (SAEs), which were graded according to National Cancer Institute Common Terminology Criteria for AEs version 4.03. Disease assessments per RECIST v1.1 [27] were performed at screening, every 8 weeks through 56 weeks and then every 12 weeks, and at the end-of-treatment visit and every 12 weeks thereafter until progression using physical examination (including skin lesion measurement), computed tomography with contrast (preferred) or magnetic resonance imaging of the head, neck, chest, abdomen, pelvis (and brain, if neurologically symptomatic). Serial blood and tumor samples were collected for PK, immunogenicity, and pharmacodynamic/biomarker analyses (Supplementary Methods).

Oleclumab and durvalumab serum concentrations were measured using a validated colorimetric enzyme-linked immunosorbent assay (ELISA) with a lower limit of quantitation of 1.00 µg/mL for oleclumab and 0.05 µg/mL for durvalumab. Pharmacokinetic parameters were estimated by noncompartmental analysis using Phoenix 64 WinNonlin 6.3 (Pharsight, Mountain View, CA). ADA responses to oleclumab and durvalumab were evaluated using a validated electrochemiluminescent immunoassay.

Flow cytometry for evaluation of total numbers and subsets of T cells was performed by Covance Central Lab Services/Lab Corp using analytically validated assays (Supplementary Methods). The concentration of soluble CD73 in human serum samples was determined using an ELISA, and whole blood samples were analyzed for circulating levels of soluble factors (Supplementary Methods). Immunohistochemistry was used to analyze matched pairs of tumor specimens at screening and during therapy (Supplementary Methods). A CD73 enzymatic assay was conducted on matched pairs of tumor specimens (Supplementary Methods).

### Statistical considerations

Up to 48 DLT-evaluable patients were to be enrolled in the escalation cohorts. Up to 280 patients (100 CRC, 100 PDAC, 80 NSCLC) were to be enrolled in the expansion cohorts. For each expansion cohort, an initial interim analysis was performed when the first 20 patients had been enrolled and followed for ≥ 16 weeks, and a second interim analysis was performed after 40 patients had been enrolled and followed for ≥ 16 weeks (Supplementary Methods).

Analysis populations are defined in Supplementary Methods. Data were summarized using descriptive statistics. Tumor response was estimated by simple proportions with confidence intervals (CI) calculated using the exact binomial method. Statistical evaluation of translational data used a 2-tailed Student t test. Statistics were performed using GraphPad Prism or R. P-values below 0.05 were considered statistically significant for tumor assessments and adjusted p-values (false discovery rate < 0.05) were considered statistically significant for whole blood assessments.

## Results

### Patient demographics and disposition

Between July 24, 2015, and August 14, 2019, 192 patients were enrolled into 4 monotherapy (*n* = 42) and 4 combination therapy (*n* = 24) escalation cohorts and the MSS-CRC (*n* = 42), PDAC (*n* = 42), and EGFRm NSCLC (*n* = 42) expansion cohorts (Supplementary Figure. 1). Of the 66 patients in the escalation cohorts, 35 had CRC and 31 had PDAC (Table 1). The majority of patients in each cohort were white, except for the NSCLC cohort, in which the majority were Asian. In the escalation cohorts, 62% of patients on monotherapy and 54% on combination therapy had received ≥ 3 prior lines of therapy; the rates were 67, 12, and 40% in the MSS-CRC, PDAC, and NSCLC expansion cohorts, respectively (Table 1). At database lock (May 24, 2021), all patients had discontinued treatment, due to disease progression (83%), withdrawal (5%), AEs (4%), death (4%), study being closed (2%), and complete response (CR; 1%).

**Table 1:** Baseline demographics and disease characteristics

	Dose-escalation phase (*n* = 66)		Expansion phase: oleclumab 40 mg/kg + durvalumab 10 mg/kg (*n* = 126)		
	Single-agent oleclumab 5–40 mg/kg (*n* = 42)	Oleclumab 5–40 mg/kg + durvalumab 10 mg/kg (*n* = 24)	MSS-CRC (*n* = 42)	PDAC (*n* = 42)	EGFRm NSCLC (*n* = 42)
Age, median (range), years	59.5 (36–81)	55 (32–71)	53 (25–80)	63.5 (32–77)	61.5 (34–83)
Sex, *n* (%)					
Male	20 (48)	9 (38)	19 (45)	16 (38)	17 (40)
Female	22 (52)	15 (63)	23 (55)	26 (62)	25 (60)
Race, *n* (%)					
White	33 (79)	21 (88)	32 (76)	37 (88)	10 (24)
Black or African American	3 (7)	2 (8)	4 (10)	1 (2)	1 (2)
Asian	2 (5)	1 (4)	5 (12)	2 (5)	30 (71)
Other	4 (10)	0	1 (2)	2 (5)	1 (2)^a^
ECOG PS, *n* (%)					
0	23 (55)	11 (46)	24 (57)	10 (24)	8 (19)
1	19 (45)	13 (54)	18 (43)	32 (76)	34 (81)
Cancer type, *n* (%)					
CRC^b^	23 (55)	12 (50)	42 (100)	0	0
PDAC	19 (45)	12 (50)	0	42 (100)	0
EGFRm NSCLC	0	0	0	0	42 (100)
Time since initial diagnosis, median (range), months	26.1 (9.6–176.5)	31.5 (5.3–78.7)	36.7 (12.1–116.4)	14.8 (4.4–90.3)	26.0 (5.5–212.1)
Number of target lesions, median (range)	2 (1–5)	2 (1–5)	3 (1–5)	2 (1–5)	2 (1–5)
Prior lines of therapy,^c^ *n* (%)					
1	2 (5)	4 (17)	1 (2)	15 (36)	9 (21)
2	14 (33)	7 (29)	13 (31)	22 (52)	16 (38)
3	16 (38)	7 (29)	12 (29)	5 (12)	11 (26)
≥ 4	10 (24)	6 (25)	16 (38)	0	6 (14)
Smoking history – yes, *n* (%)	16 (38)	8 (33)	12 (29)	10 (24)	12 (29)

ECOG PS Eastern Cooperative Oncology Group performance status, EGFRm epidermal growth factor receptor-mutant, MSI microsatellite instability, MSS-CRC microsatellite-stable colorectal cancer, NSCLC non-small-cell lung cancer, PDAC pancreatic ductal adenocarcinoma

^a^Multiple categories checked

^b^MSI status in patients with CRC was MSS for all patients with known status; unknown status in 11/23 (48%), 3/12 (25%), and 3/42 (7%) patients in the single-agent dose-escalation, combination dose-escalation, and CRC expansion cohorts, respectively

^c^Adjudicated

### Dose escalation

The DLT-evaluable population comprised 38 and 16 patients in the monotherapy and combination therapy escalation cohorts, respectively (Supplementary Figure 1). No DLTs occurred in either cohort. The oleclumab 40 mg/kg dose was the protocol-specified maximum administered dose and was used in the expansion phase based on evaluation of all available safety, PK, pharmacodynamic biomarker, and preliminary antitumor activity data in the dose-escalation phase, described below.

### Safety

The median duration of oleclumab exposure was 8.0 weeks in the monotherapy (range, 2.0–51.9) and combination therapy (range, 2.0–43.9) escalation cohorts and in the expansion cohorts combined (range, 2.0–188.1); 5 patients in the expansion cohorts (1 MSS-CRC, 1 PDAC, 3 NSCLC) received treatment for ≥ 64 weeks. Median (range) duration of exposure to durvalumab was the same as for oleclumab in the combination therapy escalation cohort and in the expansion cohorts.

#### Escalation cohorts

Common treatment-emergent AEs (TEAEs) in the escalation cohorts are summarized in Supplementary Table 1. Grade 3–4 TEAEs reported in ≥ 5% of patients in the monotherapy cohort were ascites (12%), hyperglycemia (7%), acute kidney injury, anemia, hyponatremia, and hypotension (each 5%), and in the combination therapy cohort were alanine aminotransferase (ALT) increased, aspartate aminotransferase (AST) increased, blood bilirubin increased, and pulmonary embolism (each 8%).

In the monotherapy and combination therapy escalation cohorts, respectively, 55 and 54% of patients had treatment-related AEs (TRAEs), including 7 and 21% with Grade 3–4 TRAEs (Table 2). Common TRAEs (> 10% of patients in any cohort) during escalation were fatigue (17%) in the monotherapy cohort, and fatigue (25%), AST increased, nausea, and vomiting (each 13%) in the combination therapy cohort. The only Grade 3–4 TRAE reported in ≥ 5% of patients during escalation was AST increased (8%) in the combination therapy cohort.

**Table 2:** Treatment-related adverse events

	Dose-escalation phase (*n* = 66)		Expansion phase: oleclumab 40 mg/kg + durvalumab 10 mg/kg (*n* = 126)		
TRAE^a^, *n* (%)	Single-agent oleclumab 5–40 mg/kg (*n* = 42)	Oleclumab 5–40 mg/kg + durvalumab 10 mg/kg (*n* = 24)	MSS-CRC (*n* = 42)	PDAC (*n* = 42)	EGFRm NSCLC (*n* = 42)
Any TRAE	23 (55)	13 (54)	25 (60)	24 (57)	19 (45)
Any Grade 3–4 TRAE	3 (7)	5 (21)	8 (19)	7 (17)	5 (12)
Death due to TRAE	0	0	1 (2)^d^	0	0
Any serious TRAE	0	1 (4)	2 (5)	5 (12)	3 (7)
Any TEAE leading to discontinuation^b^	2 (5)	2 (8)	3 (7)	1 (2)	2 (5)
TRAEs in > 10% of patients in any cohort^c^					
Fatigue	7 (17)	6 (25)	7 (17)	5 (12)	7 (17)
Diarrhea	0	2 (8)	5 (12)	4 (10)	2 (5)
AST increased	2 (5)	3 (13)	4 (10)	2 (5)	1 (2)
Vomiting	2 (5)	3 (13)	2 (5)	5 (12)	0
Nausea	4 (10)	3 (13)	4 (10)	0	0
Rash	1 (2)	1 (4)	3 (7)	1 (2)	5 (12)
Grade 3–4 TRAEs in ≥ 1 patient in any cohort^c^					
Aspartate aminotransferase increased	0	2 (8)	1 (2)	1 (2)	1 (2)
Hyperglycemia	1 (2)	1 (4)	0	1 (2)	0
Lipase increased	1 (2)	0	2 (5)	0	0
Alanine aminotransferase increased	0	1 (4)	1 (2)	0	0
Amylase increased	1 (2)	0	0	0	1 (2)
Blood alkaline phosphatase increased	0	0	1 (2)	1 (2)	0
Acute kidney injury	0	0	0	0	1 (2)
Anemia	0	0	0	0	1 (2)
Asthenia	0	0	0	1 (2)	0
Blood bilirubin increased	0	0	0	1 (2)	0
Blood creatinine increased	0	0	0	0	1 (2)
Colitis	0	0	0	1 (2)	0
Cytokine release syndrome	0	0	1 (2)	0	0
Dyspnea	0	0	1 (2)	0	0
Embolic stroke	0	0	0	1 (2)	0
Embolism	0	0	0	1 (2)	0
Eosinophilic fasciitis	0	0	1 (2)	0	0
Gamma-glutamyltransferase increased	1 (2)	0	0	0	0
Headache	0	1 (4)	0	0	0
Hepatitis	0	0	0	0	1 (2)
Hypertension	0	0	0	0	1 (2)
Hyponatremia	0	0	1 (2)	0	0
Immune-mediated hepatitis	0	0	0	1 (2)	0
Peripheral edema	0	0	1 (2)	0	0
Renal failure	0	0	0	0	1 (2)
Systemic inflammatory response syndrome	0	0	1 (2)	0	0
Thrombocytopenia	0	1 (4)	0	0	0
Vomiting	0	0	0	1 (2)	0
Serious TRAEs in ≥ 1 patient in any cohort^c^					
Abdominal pain	0	0	0	1 (2)	0
Acute kidney injury	0	0	0	0	1 (2)
Asthenia	0	0	0	1 (2)	0
Blood creatinine increased	0	0	0	0	1 (2)
Colitis	0	0	0	1 (2)	0
Embolic stroke	0	0	0	1 (2)	0
Embolism	0	0	0	1 (2)	0
Eosinophilic fasciitis	0	0	1 (2)	0	0
Hepatitis	0	0	0	0	1 (2)
Immune-related hepatitis	0	0	0	1 (2)	0
Pulmonary embolism	0	0	0	0	1 (2)
Renal failure	0	0	0	0	1 (2)
Systemic inflammatory response syndrome	0	0	1 (2)	0	0
Thrombocytopenia	0	1 (4)	0	0	0
Vomiting	0	0	0	1 (2)	0

*AST* aspartate aminotransferase, *EGFRm* epidermal growth factor receptor-mutant, *MSS-CRC* microsatellite-stable colorectal cancer, *NSCLC* non-small-cell lung cancer, *PDAC* pancreatic ductal adenocarcinoma, *TEAE* treatment-emergent adverse event, *TRAE* treatment-related adverse event

^a^Considered at least possibly related to oleclumab

^b^Discontinuation of oleclumab. These treatment-emergent AEs included: small intestinal obstruction in 1 patient and pulmonary embolism in 1 patient in the oleclumab 20 mg/kg monotherapy cohort; increased aspartate aminotransferase and increased blood bilirubin in 1 patient in the oleclumab 5 mg/kg + durvalumab 10 mg/kg group and increased alanine aminotransferase and increased blood alkaline phosphatase in 1 patient in the oleclumab 40 mg/kg + durvalumab 10 mg/kg group; eosinophilic fasciitis, peripheral edema, and systemic inflammatory response syndrome in 1 patient each in the MSS-CRC expansion cohort; immune-mediated hepatitis in 1 patient in the PDAC expansion cohort, and hepatitis and renal failure in 1 patient each in the EGFRm NSCLC expansion cohort

^c^TRAEs and serious TRAEs listed in order of total overall frequency across all cohorts

^d^Systemic inflammatory response syndrome

TEAEs of special interest for oleclumab occurred in 19 and 21% of patients in the monotherapy and combination therapy escalation cohorts, respectively. The most common were peripheral edema (10%) in the monotherapy cohort and peripheral edema and pulmonary embolism (each 8%) in the combination therapy cohort (Supplementary Table 2). AEs of special or possible interest for durvalumab occurred in 54% of patients in the combination therapy escalation cohort, with increased AST, increased ALT, and increased blood bilirubin (each 13%) being the most common (Supplementary Table 2).

#### Expansion cohorts

Grade 3–4 TEAEs reported in ≥ 5% of patients overall in the expansion cohorts were increased AST, blood alkaline phosphatase increased (each 6%), gamma-glutamyl transferase increased, and pleural effusion (each 5%) (Supplementary Table 1). In the MSS-CRC, PDAC, and NSCLC expansion cohorts, respectively, 60, 57, and 45% of patients reported TRAEs, including 19, 17, and 12% with Grade 3–4 TRAEs. Common TRAEs (> 5% of expansion-phase patients overall) included fatigue (15%), diarrhea (9%), rash (7%), and AST increased, pyrexia, and vomiting (each 6%). No Grade 3–4 TRAEs were reported in > 5% of patients overall in the expansion cohorts.

TEAEs of special interest for oleclumab occurred in 29, 38, and 29% of patients in the MSS-CRC, PDAC, and NSCLC expansion cohorts, respectively (Supplementary Table 2). Overall, these were most commonly peripheral edema (10%), pleural effusion (9%), and pulmonary embolism (6%). TEAEs of special or possible interest for durvalumab occurred in 55, 57, and 38% patients in the MSS-CRC, PDAC, and NSCLC expansion cohorts, respectively (Supplementary Table 2). Overall, the most common were AST increased (15%), diarrhea (14%), and ALT increased (9%).

Of all 192 enrolled patients, 5% discontinued treatment permanently due to TEAEs (Supplementary Table 1). Three patients died due to AEs, including 2 patients in the monotherapy dose-escalation cohort (small intestinal obstruction and the preferred term of ‘death’) and 1 in the CRC expansion cohort (systemic inflammatory response syndrome). A further 7 patients died on treatment due to their disease.

### Pharmacokinetics and immunogenicity

For the dose-escalation cohorts, oleclumab mean serum concentration–time profiles during cycle 1 are shown in Supplementary Figure 2 and PK parameters for oleclumab as monotherapy or in combination with durvalumab by dose level are shown Supplementary Table 3. Oleclumab serum exposures increased with increasing doses and were not affected by the addition of durvalumab. Following the first dose, oleclumab appeared to exhibit nonlinear PK at the lowest dose of 5 mg/kg, as most evident in the trough concentrations, and showed linear PK at doses of 10 mg/kg and higher. The PK exposures, as determined by C_trough_, increased in a more than dose-proportional manner from 5 to 10 mg/kg and in an approximately dose-proportional manner from 10 to 40 mg/kg. The PK parameters in the dose-expansion cohorts were similar to those in the escalation cohorts (data not shown). Mean oleclumab serum concentrations at the 20 and 40 mg/kg dose levels in both the monotherapy and combination therapy escalation cohorts were above the minimum preclinical target concentration (determined from tumor growth inhibition modeling in mice; data not shown) throughout the dosing interval, supporting selection of the 40 mg/kg dose level for the expansion phase.

Because of the Q2W dosing and the long half-life of monoclonal antibodies, terminal elimination phase PK of oleclumab were not collected and available observed oleclumab serum concentration data were insufficient for derivation of half-life. Therefore, half-life was calculated based on PK profiles simulated by a preliminary population PK model, which indicated the terminal half-life of oleclumab was 13 days at doses ≥ 10 mg/kg Q2W. The PK profile of durvalumab in combination with oleclumab was similar to that previously reported for durvalumab monotherapy (data not shown) [28].

No patients in the dose-escalation cohorts had ADAs to oleclumab at baseline or post-baseline. In the dose-escalation combination therapy cohorts, 2 of 18 assessed patients were ADA-positive for durvalumab at baseline, but none of 14 assessed patients were ADA-positive post-baseline.

In the expansion cohorts, 3 of 124 (2.4%) patients assessed were positive for ADAs to oleclumab at baseline, and 1 of 109 (0.9%) patients assessed were ADA-positive post-baseline; this patient was among those who were ADA-positive at baseline and showed persistent ADA-positive status for ≥ 16 weeks. The presence of ADAs did not alter oleclumab PK. Four of 122 (3.3%) patients assessed had ADAs to durvalumab at baseline, and 2 of 96 (2.1%) had ADAs post-baseline.

### Pharmacodynamics

**Fig. 1:**
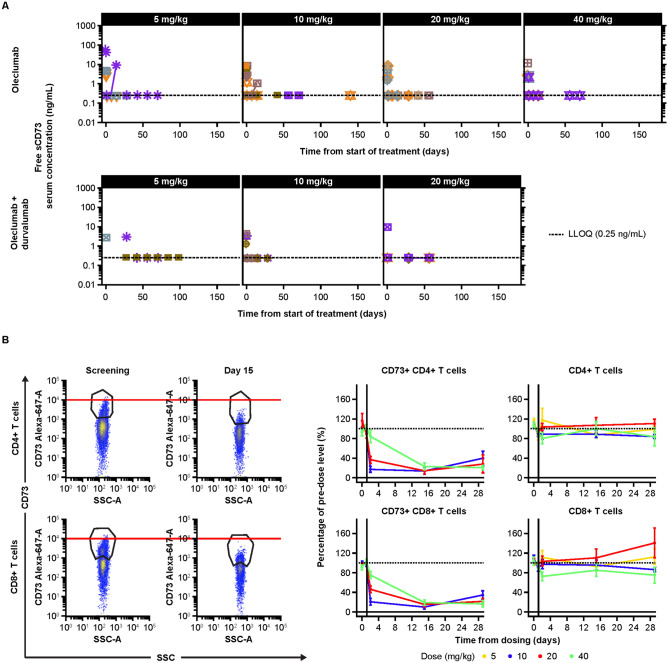
Pharmacodynamic effects of oleclumab in the periphery. **a** Sustained decreases in free soluble CD73 were observed with oleclumab monotherapy (top; 5 mg/kg, *n* = 3; 10 mg/kg, *n* = 10; 20 mg/kg, *n* = 9; 40 mg/kg, *n* = 6) or in combination with durvalumab (bottom; 5 mg/kg, *n* = 3; 10 mg/kg, *n* = 3; 20 mg/kg, *n* = 3) in the escalation phase; **b** oleclumab monotherapy decreased CD73 cell surface expression, as measured by mean fluorescent intensity, and percentage of CD73+, CD4+ and CD8+ cells across all doses (baseline = 100%), without a concomitant decrease in total absolute numbers of CD4+ and CD8+ cells. Oleclumab monotherapy: 5 mg/kg, *n* = 3; 10 mg/kg, *n* = 11; 20 mg/kg, *n* = 12; 40 mg/kg, *n* = 15. CD73 cluster of differentiation 73, LLOQ lower limit of quantitation, SSC side scatter

A sustained decrease in free soluble CD73 was observed with oleclumab both as monotherapy and in combination with durvalumab in the escalation phase (Fig. 1a). In the blood, sustained decreases of CD73 on peripheral T lymphocytes were also noted, consistent with the internalization of the bound CD73 protein (Fig. 1b).

**Fig. 2:**
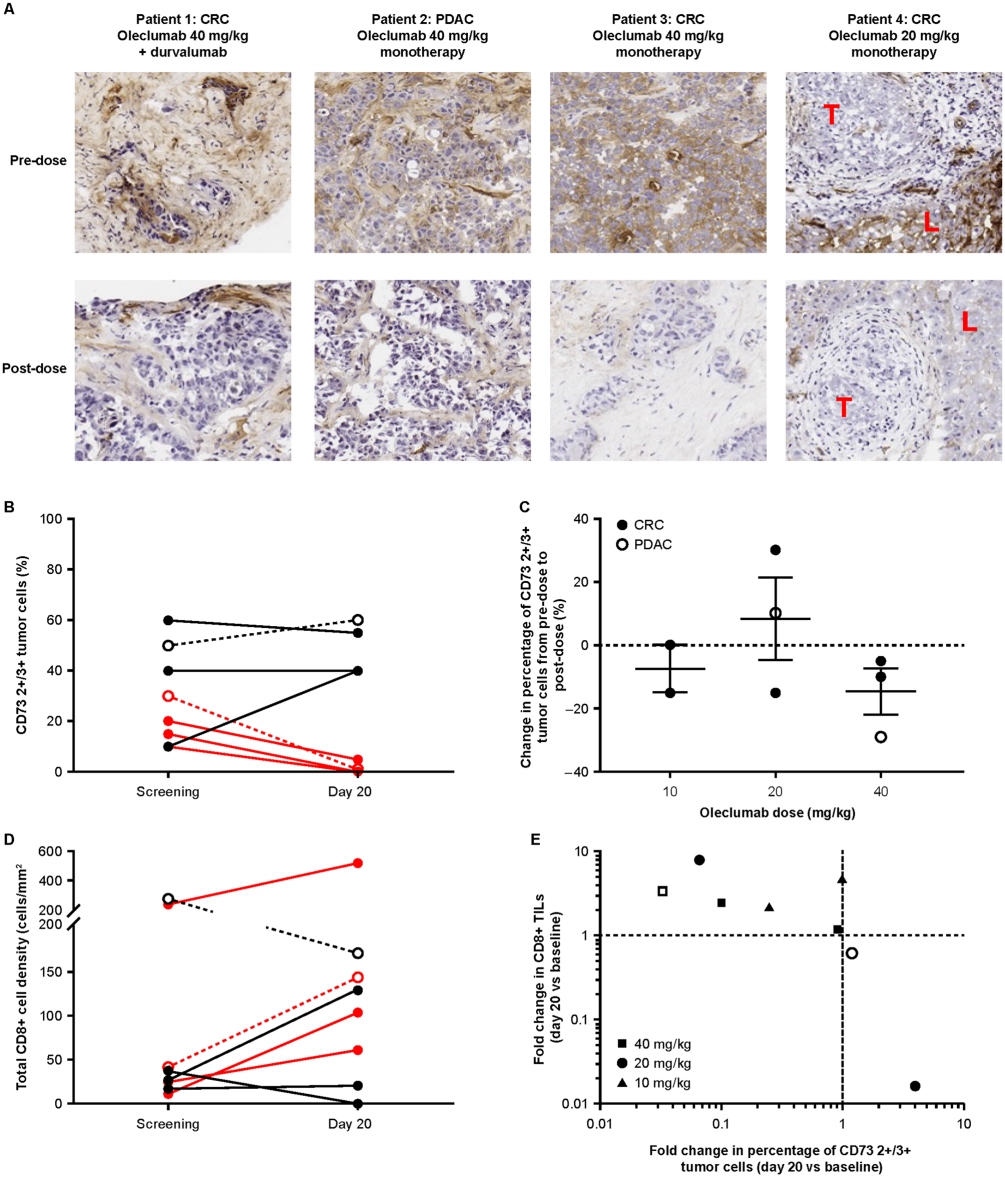
Pharmacodynamic effects of oleclumab in tumor tissue. **a** Decrease in CD73 enzymatic activity 20 days after treatment initiation as demonstrated by an in situ enzymatic assay. Patient 4 had no tumor cell CD73 expression or enzymatic activity at baseline but had CD73 enzymatic activity in adjacent liver tissue prior to treatment that decreased following treatment. Representative images are shown at 20X magnification. With this method, enzymatic activity leads to a brown coloring. Patients with CRC or PDAC were treated with oleclumab monotherapy and their tumors were assessed for CD73 and CD8 expression. **b** Change of 2+ /3+ CD73 staining in tumor cells from screening to 20 days after oleclumab initiation. **c** Change of 2+ /3+ CD73 staining in tumor cells from screening to 20 days after treatment initiation by oleclumab dose. **d** Quantification of total CD8+ cells/mm^2^ at screening and 20 days after oleclumab treatment and **e** correlation of the fold change in CD8+ cells with the fold change in the percentage of 2+ /3+ CD73+ tumor cells 20 days after treatment initiation. Panels **b-e** show patients who had baseline CD73 expression of ≥ 10% on tumor cells. In panels **b** and **d**, red lines represent patients who had ≥ twofold decreases from screening in CD73 levels. In panels **b**, **c,** and **d**, filled symbols (and solid lines in panels **b** and **d**) represent patients with CRC and open symbols (and dashed lines in panels **b** and **d**) represent patients with PDAC. CD73 cluster of differentiation 73, CRC colorectal cancer, L liver, LLOQ lower limit of quantitation, PDAC pancreatic ductal adenocarcinoma, T tumor, TILs tumor infiltrating lymphocytes

In the tumor, an ectonucleotidase enzymatic activity assay [29] of tissue oleclumab penetrance demonstrated a decrease in CD73 enzymatic activity 20 days after the first oleclumab dose in tissues from all 4 patients with evaluable paired biopsies who received oleclumab monotherapy (*n* = 3) or oleclumab in combination with durvalumab (*n* = 1) in the escalation phase (Fig. 2a). In 3 of 4 patients with CD73 expression on tumor cells, cells in the post-treatment sample had lower enzymatic activity than those in the pretreatment sample. One patient with liver metastases had no staining of CD73 in tumor cells but the adjacent liver tissue was positive for CD73 stain and enzymatic activity. After oleclumab treatment, enzymatic activity was decreased in the liver sample while the tumor remained negative (Fig. 2a). Additionally, immunohistochemistry showed a decrease in CD73+ tumor cells in all 3 patients treated with oleclumab 40 mg/kg who expressed CD73 at baseline (Fig. 2b, c). A decrease in CD73, independent of dose, was associated with an increase in CD8+ T cells after oleclumab monotherapy (Fig. 2d, e). These findings supported selection of the 40 mg/kg dose level for the expansion phase.

**Fig. 3:**
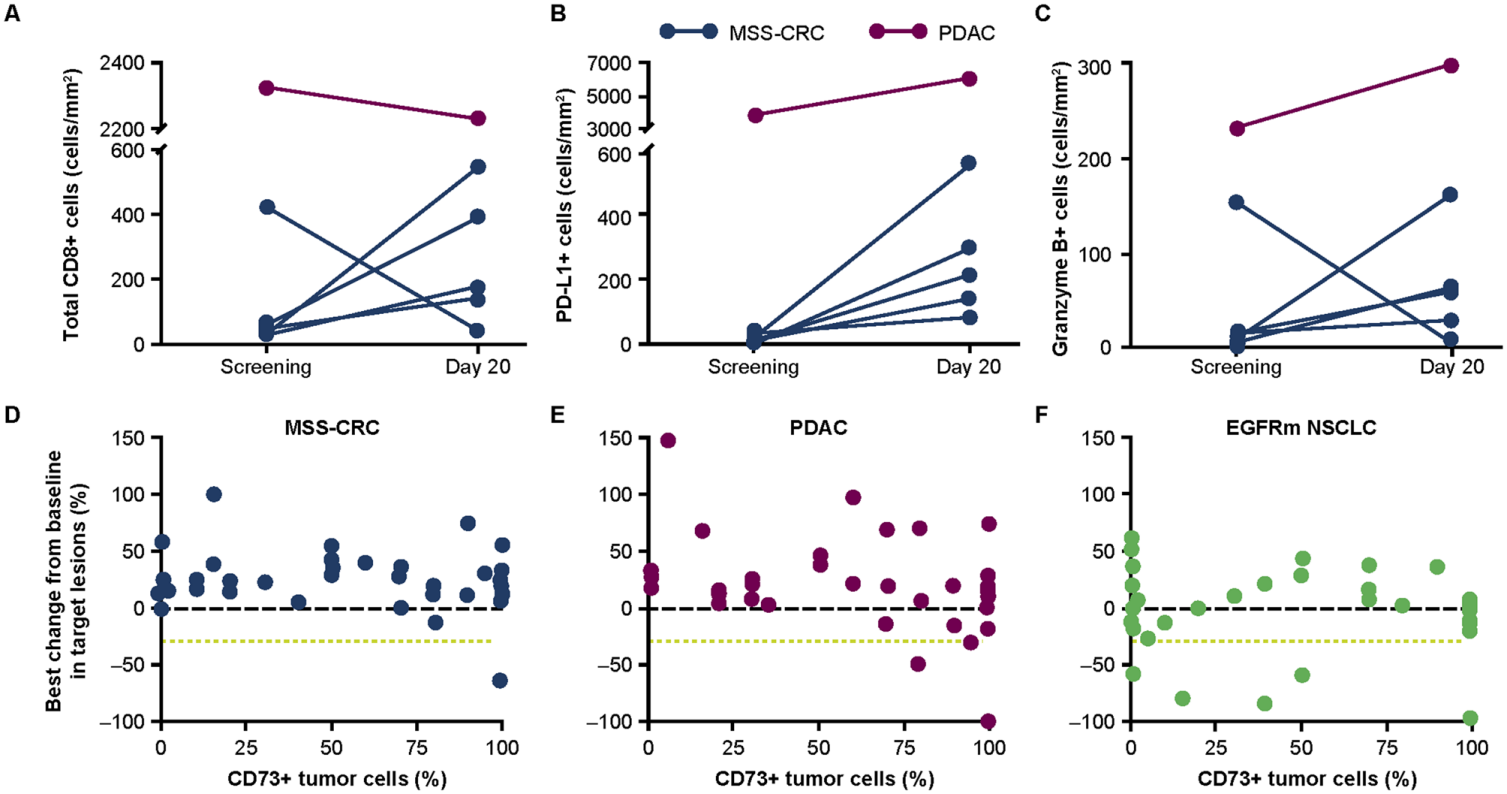
Biomarkers in patients who received oleclumab plus durvalumab. The pharmacodynamics of **a** CD8+ T cells, **b** PD-L1+ tumor cells, and **c** granzyme B+ cells in tumors of 6 patients with MSS-CRC (blue symbols and lines) or PDAC (purple symbols and lines) who had matched biopsies. The relationship between baseline tumoral CD73 expression and best overall response (percent change from baseline in target lesions) in patients with **d** MSS-CRC, **e** PDAC, and **f** EGFRm NSCLC from the dose-expansion cohorts. CD73 cluster of differentiation 73, CRC colorectal cancer, EGFRm epidermal growth factor receptor-mutant, MSS microsatellite-stable, NSCLC non-small-cell lung cancer, PDAC pancreatic ductal adenocarcinoma, PD-L1 programmed cell death ligand-1, T tumor

Among 6 patients with matched biopsies who received combination therapy, 4 had increases in CD8+ T cells, 6 had increases in PD-L1+ cells, and 5 had increases in granzyme B+ cells (Fig. 3a–c).

### Antitumor activity

No ORs were seen in the dose-escalation cohorts; the best percentage changes in target lesion size from baseline are shown in Supplementary Figure 3, including 1 patient in the oleclumab 40 mg/kg combination therapy group who had a > 30% decrease in target lesion size.

Stable disease (SD) ≥ 8 weeks was observed in 14.3% of patients in the monotherapy dose-escalation cohorts and in 8.3% of patients in the combination therapy dose-escalation cohorts. Median PFS was 1.8 months in both the monotherapy and combination therapy dose-escalation cohorts, with respective 6-month PFS rates of 3.3 and 4.5%. Median OS was 6.1 months in the monotherapy cohort and 5.6 months in the combination therapy cohort, with 9-month OS rates of 42.8 and 35.6%, respectively.

**Fig. 4:**
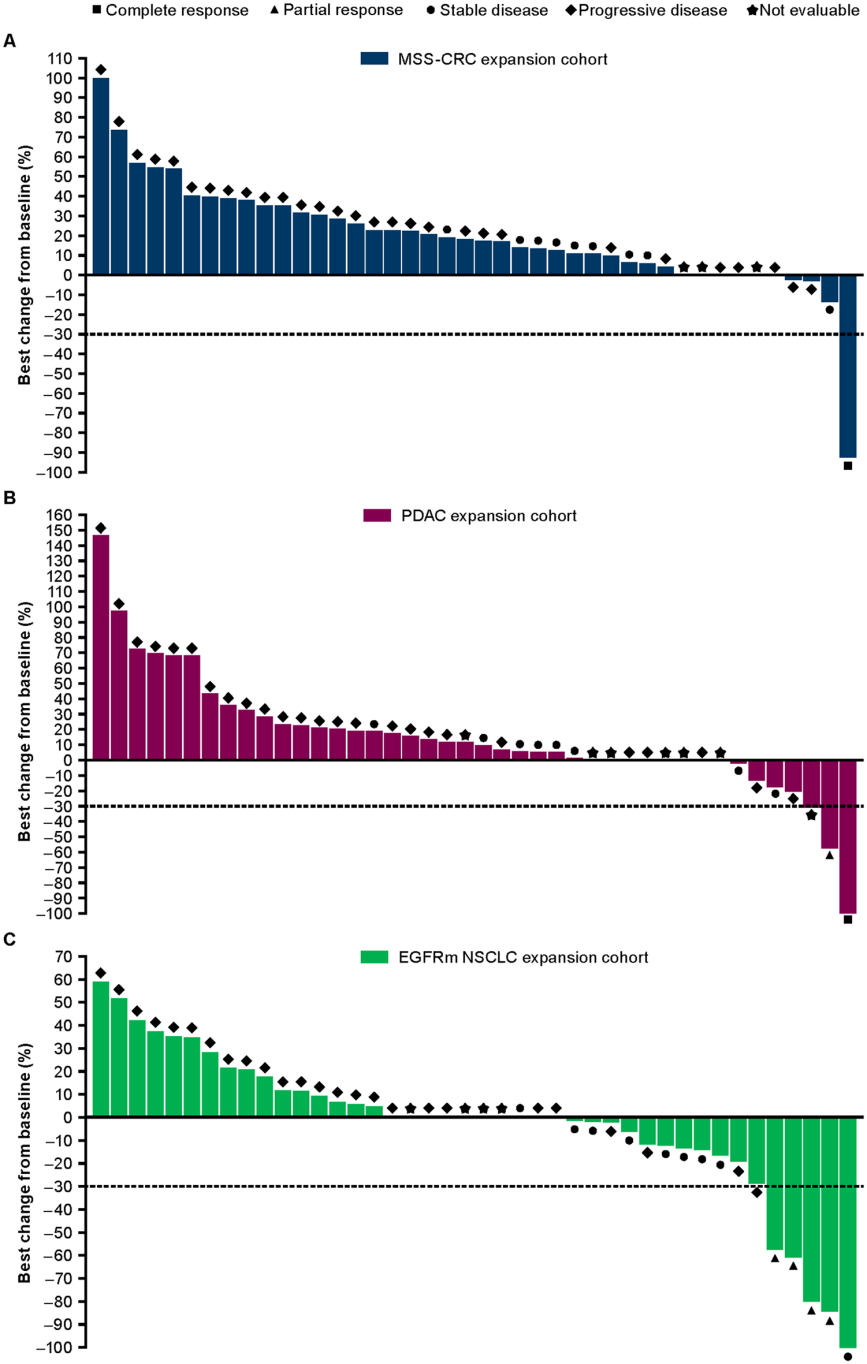
Best percentage change from baseline in target lesions in the **a** MSS-CRC, **b** PDAC, and **c** EGFRm NSCLC expansion cohorts. CRC colorectal cancer, EGFRm epidermal growth factor receptor-mutant, MSS-CRC microsatellite-stable colorectal cancer, PDAC pancreatic ductal adenocarcinoma

In the MSS-CRC expansion cohort, 1 patient (2.4%), a 38-year-old female who was a previous smoker and had received 4 prior chemotherapy regimens, had a CR following an initial partial response (PR), with a DoR of 36.2 months (Fig. 4a). An additional 9 patients (21.4%) had SD ≥ 8 weeks. Overall, DC was ≥ 24 weeks in 2 (4.8%) patients with MSS-CRC.

In the PDAC expansion cohort, 2 patients (4.8%) had ORs, including 1 CR in a 58-year-old female who had had a PR to her one prior systemic chemotherapy regimen, and 1 PR in a 67-year-old female with confirmed MSS PDAC who had had a PR to her one prior chemotherapy regimen; both responses were ongoing at database lock, with DoRs of 30.4 and 37.0 months, respectively (Fig. 4b). An additional 8 patients (19.0%) had SD ≥ 8 weeks. Overall, DC was ≥ 24 weeks in 5 (11.9%) patients.

In the EGFRm NSCLC expansion cohort, 4 patients (9.5%) had PRs, with a median DoR of 14.8 months (range, 5.6–24.0; 1 response ongoing) (Fig. 4c). An additional 9 patients (21.4%) had SD ≥ 8 weeks. Overall, DC was ≥ 24 weeks in 6 (14.3%) patients.

Baseline tumor CD73 expression and association with clinical response is summarized in Fig. 3d–f. In the 3 patients with MSS-CRC or PDAC who had a confirmed response, ≥ 80% of tumor cells expressed CD73. There was no association between CD73 expression and response in patients with EGFRm NSCLC, and nor was there an association between PD-L1 tumor cell expression and response in these patients (Supplementary Figure 4).

In all 3 expansion cohorts, the median PFS was 1.8 months; 6-month PFS rates in the MSS-CRC, PDAC, and NSCLC cohorts were 5.4%, 13.2%, and 16.0%, respectively. Median OS was 7.0 months in the MSS-CRC cohort, 6.3 months in the PDAC cohort, and 11.3 months in the NSCLC cohort, with 9-month OS rates of 31.2%, 31.6%, and 57.0%, respectively.

## Discussion

This phase I, first-in-human study of the anti-CD73 monoclonal antibody oleclumab in patients with advanced solid tumors showed a manageable safety profile when administered alone or in combination with durvalumab, with no DLTs and the maximum investigated dose of oleclumab, 40 mg/kg, being feasible for use in combination with standard-dose durvalumab. Oleclumab exposure profile was consistent with those of other monoclonal antibodies and supportive of the Q2W administration schedule, while pharmacodynamic studies showed activity consistent with the mechanism of action of oleclumab, namely inhibition of the enzymatic activity of CD73 and reduction in cell surface CD73 levels. There were isolated cases of durable ORs, predominantly in EGFRm NSCLC, with the combination of oleclumab and durvalumab, consistent with preclinical findings [9, 23, 25].

Prolonged exposure to oleclumab in combination with durvalumab was tolerable, with 5 patients receiving treatment for > 1 year. Although just over half the patients had ≥ 1 TRAE, the incidence of Grade 3–4 TRAEs was < 20% in all expansion cohorts treated at the maximum dose of oleclumab, and there was a low overall rate (5%) of treatment discontinuation due to TEAEs. Three (2%) patients died due to TEAEs. Common TEAEs and TRAEs did not include any unexpected toxicities, and the safety profile of combination therapy reflected the anticipated AEs associated with durvalumab [30–33] and monoclonal antibody therapy more generally [34].

Oleclumab exposure was dose-dependent across the 4 dose levels evaluated and was not impacted by concomitant use of durvalumab. Oleclumab PK appeared linear at doses ≥ 10 mg/kg Q2W and were similar to those of other human IgG1 monoclonal antibodies, with an estimated half-life of 13 days [35]. Immunogenicity to both oleclumab and durvalumab was rare, consistent with the low rates of ADA responses to durvalumab [28].

Target engagement in the periphery was demonstrated by a sustained decrease in free soluble CD73 with oleclumab, suggesting saturation of binding to soluble CD73. Flow cytometry analysis of peripheral blood mononuclear cells showed decreased CD73 levels on both CD4+ and CD8+ T lymphocytes, consistent with the internalization of the bound CD73 protein [25]. Oleclumab inhibited CD73 enzymatic activity and decreased CD73 levels on tumor cell surfaces, consistent with its mechanism of action. Four of 8 patients treated with oleclumab monotherapy showed a twofold decrease of CD73 expression on treatment, with a concomitant ≥ twofold increase in CD8+ T cell infiltration in tumor tissues, providing preliminary evidence of immunomodulation by oleclumab in patients with MSS-CRC and PDAC. In the MSS-CRC and PDAC expansion cohorts, 4 of 6 patients with matched biopsies had an increase in CD8+ T cell infiltration in tumors along with increased PD-L1 expression and granzyme B positive cells, further reinforcing the immunomodulatory effect of this combination in tumor types that are generally considered immune quiescent. As none of these patients obtained an OR in the context of these immunologic changes, there is a need for additional understanding of the quality of these immune cells as well as additional strategies to modulate the immunosuppressive nature of the tumor microenvironment.

Aggregated safety, PK, pharmacodynamic, and anti-tumor activity data informed the selection of the oleclumab 40 mg/kg dose level for the combination expansion phase. Antitumor activity with the combination of oleclumab and durvalumab was promising in patients with EGFRm NSCLC, and ORs seen in patients in the expansion cohorts were associated with durable disease control of up to ~ 3 years, including in tumors typically resistant to immunotherapy. In the phase II ATLANTIC study of single-agent durvalumab as third-line or later treatment for advanced NSCLC, OR rate and DC rate at 6 months in 102 patients with EGFRm, *ALK*-positive disease, regardless of PD-L1 expression, were 9.8% and 16.7%, respectively, and median DoR was 7.4–7.9 months [36], indicating limited antitumor activity with durvalumab monotherapy in this setting. However, in the phase II, randomized COAST study, the combination of oleclumab and durvalumab improved OR rate and PFS versus durvalumab alone in patients with locally advanced, unresectable, Stage III NSCLC who had not progressed following concurrent chemoradiotherapy [37].

Exploratory analysis of CD73 expression on tumor cells in baseline samples with best overall response suggests that high expression may be associated with clinical benefit in patients with MSS-CRC and PDAC. In the EGFRm NSCLC expansion cohort there was no association between response and CD73 expression in the archival tissue assessed. However, the baseline archival sample time-point was not pre-specified or controlled, which may have led to additional variability arising due to the potential effect of prior treatment (such as an EGFR inhibitor) on CD73 expression [9]. CD73 should be further evaluated as a potential biomarker of clinical benefit in studies evaluating adenosine-pathway blocking agents in various combinations, including ongoing studies in PDAC, CRC, NSCLC, and triple-negative breast cancer. Based on the mechanistic rationale, preclinical evidence, and promising initial data, several studies utilizing oleclumab plus durvalumab or with standard of care chemotherapies are ongoing in a range of tumor types, including studies in NSCLC (MAGELLAN, NCT03819465; NeoCOAST-2, NCT05061550; PACIFIC-9, NCT05221840), CRC (COLUMBIA-1, NCT04068610), triple-negative breast cancer (SYNERGY, NCT03616886; BEGONIA, NCT03742102), luminal B breast cancer (Neo-CheckRay, NCT03875573), and sarcoma (NCT04668300).

### Supplementary Information

Below is the link to the electronic supplementary material.

Supplementary file1 (DOCX 372 kb)

## Data Availability

Data underlying the findings described in this manuscript may be obtained in accordance with AstraZeneca’s data sharing policy described at https://astrazenecagrouptrials.pharmacm.com/ST/Submission/Disclosure. Data for studies directly listed on Vivli can be requested through Vivli at www.vivli.org. Data for studies not listed on Vivli could be requested through Vivli at https://vivli.org/members/enquiries-about-studies-not-listed-on-the-vivli-platform/. AstraZeneca’s Vivli member page is also available outlining further details: https://vivli.org/ourmember/astrazeneca/.

## Abbreviations

ADA: Antidrug antibodies
AE: Adverse events
ALT: Alanine aminotransferase
AST: Aspartate aminotransferase
CD73: Cluster of differentiation 73
CI: Confidence interval
CR: Complete response
CRC: Colorectal cancer
DC: Disease control
DLT: Dose-limiting toxicity
DoR: Duration of response
EGFRm: Epidermal growth factor receptor-mutant
ELISA: Enzyme-linked immunosorbent assay
IV: Intravenous
LLOQ: Lower limit of quantitation
MSS: Microsatellite-stable
MTD: Maximum tolerated dose
NSCLC: Non-small-cell lung cancer
OR: Objective response
OS: Overall survival
PD: Programmed cell death
PDAC: Pancreatic ductal adenocarcinoma
PD-1: Programmed cell death-1
PD-L1: Programmed cell death ligand-1
PFS: Progression-free survival
PK: Pharmacokinetics
PR: Partial response
Q2W: Every 2 weeks
RECIST: Response evaluation criteria in solid tumors
SAE: Serious AEs
SD: Stable disease
TEAEs: Treatment-emergent AEs
TRAEs: Treatment-related AEs
