# Supplementary material for: First-in-human study of oleclumab, a potent, selective anti-CD73 monoclonal antibody, alone or in combination with durvalumab in patients with advanced solid tumors
Source: Cancer Immunol Immunother. 2023 Apr 5;72(7):2443–58. doi: 10.1007/s00262-023-03430-6 (PMC10264501; doi:10.1007/s00262-023-03430-6)

**Cancer Immunology, Immunotherapy (submitted in 2022) – Johanna Bendell et al.**

**SUPPLEMENTARY MATERIAL**

**Supplementary Methods**

**Additional inclusion criteria**

Patients were required to have adequate organ function in terms of hematologic, renal, and hepatic function. Adequate hematologic function was defined as: an absolute neutrophil count ≥1.5 × 10^9^/L (1,500/mm^3^), a platelet count ≥75 × 10^9^/L (75,000/mm^3^), hemoglobin ≥9.0 g/dL, and prothrombin time-international normalized ratio and partial thromboplastin time ≤1.5 × the upper limit of normal (ULN), all without growth factor or transfusion support within 14 days prior to screening. Patients also had to be likely, in the opinion of the investigator, to complete ≥56 days of treatment. All patients required ≥1 lesion that was measurable using Response Evaluation Criteria in Solid Tumors (RECIST) v1.1 guidelines [24]; i.e., a previously irradiated lesion could be considered a target lesion if the lesion was well defined, measurable per RECIST, and had clearly progressed; patients undergoing tumor biopsies must have had additional non-target lesions that could be biopsied at acceptable risk as judged by the investigator, or if no other lesion was suitable for biopsy then the RECIST target lesion used for biopsy must have been ≥2 cm in longest diameter.

In the expansion phase, patients with microsatellite-stable (MSS)-colorectal cancer (CRC) must have received 2–4 prior lines of systemic therapy in the metastatic setting, including regimens containing a fluoropyrimidine such as 5-fluorouracil or capecitabine, oxaliplatin, and irinotecan unless contraindicated. They must not have had defective DNA mismatch repair (i.e., they had to have MSS-CRC), documented by testing. Defective DNA mismatch repair was defined by either: high-frequency microsatellite instability (MSI) with changes detected in ≥2 panels of microsatellite markers (for example, BAT-25, BAT-26, NR-21, NR-24, or MONO-27); or immunohistochemical analysis demonstrating absence of protein expression of MLH1, MSH2, MSH6, and/or PMS2. Patients with KRAS mutation (e.g., exon 2, codon 12 or 13) were eligible. A minimum of 10 patients were required to consent to undergo paired pretreatment and on-treatment tumor biopsies. Patients with pancreatic ductal adenocarcinoma (PDAC) must have received 1–2 prior lines of systemic therapy in the metastatic setting. Patients with non-small-cell lung cancer (NSCLC) required disease with an epidermal growth factor receptor (EGFR) mutation known to be associated with EGFR tyrosine kinase inhibitor (TKI) sensitivity, including G719X, exon 19 deletion, L858R, and L861Q; they must have received 1–4 prior lines of therapy, including investigational therapy, in the metastatic setting, and must have received an approved EGFR TKI and then clinically or radiologically progressed or have been intolerant to this treatment. For all expansion-phase cohorts, prior chemotherapy received in the neoadjuvant or adjuvant settings was only considered a line of therapy in the metastatic setting if recurrent disease developed within 6 months of completing therapy.

**Exclusion criteria**

Patients were ineligible if they had a history of severe drug allergies or anaphylaxis to ≥2 food products or medicines; cardiac or peripheral vascular disease (myocardial infarction in prior 12 months, history of stroke or transient ischemic attack requiring medical therapy, New York Heart Association Class ≥3 congestive heart failure); Grade ≥3 edema; Grade ≥3 thromboembolic events in the prior 12 months or thromboembolic event of any grade with ongoing symptoms; active tuberculosis; active or prior documented autoimmune disorders; untreated central nervous system metastatic disease, leptomeningeal disease, or cord compression; history of primary immunodeficiency or solid organ transplantation; or known positivity for human immunodeficiency virus (HIV), chronic or active hepatitis B, or active hepatitis A or C.

Patients receiving any concurrent chemotherapy, immunotherapy, or biologic or hormonal therapy for cancer treatment were excluded, as were patients who had received prior treatment with tumor necrosis factor receptor superfamily agonists including OX40, CD27, CD137 (4-1BB), and CD357 (glucocorticoid-induced tumor necrosis factor receptor family-related protein). Additionally, patients with EGFR-mutant (EGFRm) NSCLC must not have had prior exposure to any immunotherapy (including anti-CTLA-4, anti-PD-1, or anti-PD-L1 monoclonal antibodies, although monotherapy tumor vaccine was allowed), while patients with CRC or PDAC who had received prior therapy with regimens containing CTLA-4, PD-L1, or PD-1 antagonists were not permitted to enroll unless their last dose had been administered at least 28 days prior to the planned first dose of oleclumab/durvalumab; they had not experienced a toxicity that led to permanent discontinuation of their prior immunotherapy; all adverse events (AEs) on prior immunotherapy had resolved to Grade ≤1 or baseline prior to screening; and they had not experienced a treatment-related Grade ≥3 AE or neurologic or ocular AE of any grade that was deemed to be related to the prior immunotherapy. Patients were also excluded if they had received any conventional or investigational anticancer therapy or any live, attenuated vaccine within 28 days before the first dose of oleclumab or an EGFR TKI or immunosuppressive medication within 14 days prior to the first dose of oleclumab.

**Definition of dose-limiting toxicity**

Dose-limiting toxicities (DLTs) were evaluated during the dose-escalation phase from the time of the first dose of oleclumab until the planned administration of the third dose. DLTs were defined as any of the following during the DLT-evaluation period:

- Any Grade ≥3 treatment-related toxicity
- Any Grade 4 immune-mediated AE (imAE)
- Any Grade ≥3 colitis
- Any Grade 3 or 4 non-infectious pneumonitis, irrespective of duration
- Any Grade 3 imAE, excluding colitis or pneumonitis, that did not downgrade to Grade ≤2 within 3 days after onset, despite maximal medical supportive care including systemic corticosteroids, or did not downgrade to Grade ≤1 or baseline within 14 days
- Liver transaminase elevation ≥5 × but ≤8 × ULN that did not downgrade to Grade 2 within 5 days after onset with optimal medical management, including systemic corticosteroids
  - Transaminase elevation >8 × ULN or total bilirubin (TBL) >5 × ULN was considered a DLT regardless of duration or reversibility
- Any increase in aspartate aminotransferase or alanine aminotransferase >3 × ULN and concurrent increase in TBL >2 × ULN

The following were not included within the definition of DLT:

- Grade 3 fatigue for ≤7 days
- Grade 3 endocrine disorder (thyroid, pituitary, and/or adrenal insufficiency) that is managed with or without systemic corticosteroid therapy and/or hormone replacement therapy
- Grade 3 inflammatory reaction attributed to a local antitumor response (e.g., inflammatory reaction at sites of metastatic disease, lymph nodes, etc) that resolved to Grade ≤1 within 30 days
- Concurrent vitiligo or alopecia of any AE grade
- Grade 3 infusion-related reaction (first occurrence and in the absence of steroid prophylaxis) that resolved within 6 hours with appropriate clinical management
- Grade 3 neutropenia that was not associated with fever or systemic infection and improved by at least 1 grade within 3 days
- Grade 3 or 4 lymphopenia (unless clinically significant)
- Grade 3 thrombocytopenia that was not associated with clinically significant bleeding that required medical intervention, and improved by at least 1 grade within 7 days
- Isolated Grade 3 electrolyte abnormalities that were not associated with clinical signs or symptoms and were reversed with appropriate maximal medical intervention within 3 days
- Grade 3 fever lasting ≤24 hours with or without medical therapy and was not considered a serious AE

**Blood and tumor sample collection for pharmacokinetic, immunogenicity, and pharmacodynamic/biomarker analyses**

In protocol amendment 1 and 2, serum samples for evaluation of oleclumab pharmacokinetics were collected predose and at 10 minutes, 2 hours, and 4–6 hours after the end of infusion on day 1, anytime on day 2, day 3, and day 8, predose and 10 minutes after the end of infusion on day 15, and every 2 weeks until the end of treatment. Starting from protocol amendment 3, serum samples for evaluation of oleclumab pharmacokinetics were collected predose and 10 minutes after the end of infusion on days 1 (additional sample collected 2 hours after the end of infusion), 15, 29, and 57, then every 8 weeks through 56 weeks, and then every 12 weeks. Predose serum samples for evaluation of durvalumab pharmacokinetics were collected at the same time points as those for evaluation of oleclumab pharmacokinetics; postdose serum samples were collected at 10 minutes post end of infusion only on days 1, 15, and 29. In protocol amendment 1 and 2, serum samples for evaluation of antidrug antibodies (ADAs) to oleclumab and durvalumab were collected predose on days 1, 29, 57, 85, and 113, at the end of treatment, and at the 30-day follow-up; in addition serum samples for oleclumab ADAs were collected every 4 weeks after day 113. Starting from protocol amendment 3, serum samples for evaluation of ADAs to oleclumab and durvalumab were collected predose on days 1, 29, and 57, with further samples taken at the end of treatment and the 30-day follow-up for ADAs to oleclumab and every 12 weeks in follow-up for ADAs to durvalumab. Whole blood samples for immunophenotyping were collected on days 1, 15, and 29; serum samples for soluble cluster of differentiation (CD73) assay were collected on days 1, 15, 29, 57, and every 8 weeks thereafter through 56 weeks.

All patients required an archival tumor sample <3 years old at screening or were required to give consent and undergo tumor biopsy. Pretreatment biopsies were required for evaluation of CD73 expression at screening, where applicable. Patients in the dose-escalation cohorts, at least 10 patients in the CRC expansion cohort, and all patients in the PDAC and NSCLC expansion cohorts were encouraged to provide consent for paired pretreatment and on-treatment (day 20 ± 5) tumor biopsies where clinically feasible as determined by the treating physician.

**Flow cytometry methodology**

Whole blood samples were collected in CytoChex BCT tubes (Streck), shipped to central laboratory sites, and stained (within 78 hours of collection) with antibodies to CD3 AF700 (Clone UCHT1, Miltenyi Biotec), CD4 BV510 (Clone SK3, BD), CD45 PerCP (Clone 2D1, BD), CD8 V450 (Clone RPA-T8, BD), CD45 and CD73 AF647 clone 2C5 (proprietary to AstraZeneca), CD25-PE (Clone 2A3, BD), CD127 FITC (Clone MB15-18C9 Miltenyi Biotec), and CD19 BV605 (Clone SJ25C1, BD). Cells were acquired on BD FACSCanto™ instruments and analyzed in WinList7.0 (Verity Software).

**Ligand binding analysis for soluble CD73**

The ligand binding analysis for soluble CD73 comprised a stepwise format in which wash steps followed each incubation. A human anti-CD73 monoclonal antibody (mAb) coated to a microtiter plate was used to capture free soluble CD73 in calibration samples, quality controls, and serum samples. Any bound soluble CD73 was then detected using a horseradish peroxidase (HRP)-labeled anti-CD73 mAb (non-competing with capture mAb). TMB (3,3′,5,5′-tetramethylbenzidine) was used as the substrate for the colorimetric reaction to quantitatively measure the binding complex; chromogenic color development was directly related to levels of soluble CD73 in samples. Once the procedure had been stopped with acid, the plate was read on a spectrophotometer at 450 nm, and data were analyzed with SoftMax® Pro (SMP), version 5.4. Data were fitted using a 5-parameter logistic function with no weighting factor, and concentrations of soluble CD73 in samples were measured by interpolation from the standard curve. The quantitative range of this assay was 0.25 ng/mL (LLOQ) to 15 ng/mL (upper limit of quantitation).

**Immunohistochemical analysis**

Immunohistochemistry was used to analyze matched pairs of tumor specimens collected at screening and during therapy, with the assay validated under College of American Pathologists guidelines and performed in a Clinical Laboratory Improvement Amendments (CLIA)-certified laboratory. Use of the assay to determine eligibility based on CD73-positivity at screening in a subset of patients in the expansion cohort was not considered a significant risk to the patients, and therefore no Investigational Device Exemption (IDE) was required for this purpose. Fifteen patients in the oleclumab monotherapy dose-escalation cohorts and 6 patients treated with oleclumab plus durvalumab had evaluable pretreatment and post-treatment samples for immunohistochemistry assessment. Of the 15 patients in the monotherapy cohorts, 8 had CD73 expression ≥10% on tumor cells at the pretreatment timepoint. For the CD73 enzymatic assay, 4 patients had evaluable tumor in pre and on-treatment frozen tissue sections.

A pathologist examined a hematoxylin and eosin (H&E)-stained slide from each tissue block for the presence of tumor. Sections of 4 μm thickness were cut from a representative tumor block selected from each patient/sample for immunohistochemistry analysis; CD73, CD8 and granzyme B analyses were performed using the Ventana Discovery system, and PD-L1 analysis was performed using a Ventana Benchmark automated staining instrument (Ventana Medical Systems, Tucson, AZ). The primary antibodies were CD73 (clone EPR6115, Abcam), CD8 (clone SP239, Ventana), granzyme B (EPR8260, Abcam), and PD-L1 (SP263, Ventana) using previously optimized conditions (1). All stained slides were converted into high-resolution digital images of the whole section (e-slide) using Aperio AT Turbo or Aperio XT scanners and a 20X objective. Digital images were manually annotated by a pathologist to designate tumor area. Marker quantification was performed by Definiens AG (Munich, Germany) image analysis using Definiens Developer™ software employing customized algorithms for CD8 and was reported as marker-positive cells/mm^2^ of tumor area. CD73 was reported as a percentage of positive tumor cells expressing 2+/3+ CD73 intensity as scored by a pathologist.

**CD73 enzymatic assay methods**

Matched pairs of tumor specimens were obtained at screening and during therapy (Day 22 ± 1) and snap-frozen. Frozen tumor samples were embedded in Optimal Cutting Temperature Compound and maintained at -80°C. Samples were cryosectioned at 5 µm and air dried at room temperature. Slides were fixed in acetone at -20°C for 10 minutes before being immersed in 10% neutral buffered formalin for 2 minutes. Slides were washed in phosphate buffered saline (PBS) 3 times for 2 minutes before being incubated for 15 minutes in 2 mL/slide of 50 mM tris maleate, 2 mM calcium chloride, and 250 mM sucrose in pure water adjusted to pH 7.4 (solution 1). Excess solution was removed, and slides were incubated in 2 mL/slide of 50 mM tris maleate, 2 mM calcium chloride, and 250 mM sucrose in pure water adjusted to pH 7.4 + 1 mM AMP, 5 mM manganese chloride, 2 mM lead II nitrate, 2.5% Dextran T200, and 2.5 mM levamisole hydrochloride for 1 hour at 37°C. Slides were then washed in PBS before incubation in 1% ammonium sulfide and water for 1 minute. The solution was then removed, and the slides were immersed in 3 changes of water to stop the reaction. The slides were then counterstained with hematoxylin, dehydrated and permanently cover-slipped. Tumor and adjacent liver tissue (if present) were assessed by a pathologist.

**Statistical considerations**

For each of the MSS-CRC, PDAC, and NSCLC expansion cohorts, an initial interim analysis was to be performed when the first 20 patients had been enrolled and followed for ≥16 weeks. Further enrollment to each individual cohort was planned to proceed only if ≥2 of the first 20 patients experienced an objective response (OR) per RECIST v1.1. While only 1 of the first 21 patients with MSS-CRC had an OR, based on comparable activity to standard-of-care options for ≥3-line treatment of CRC and promising preliminary durability of OR and stable disease, the sponsor and investigators decided to proceed with enrollment to further explore the safety and preliminary antitumor activity of oleclumab plus durvalumab in this setting, with revised inclusion criteria specifying ≥2 prior lines of therapies in this cohort.

A second interim analysis in each of the expansion cohorts was to be performed after 40 patients had been enrolled and followed for ≥16 weeks. Further enrollment to the individual cohorts was dependent on the following criteria:

- MSS-CRC cohort: enrollment of up to 100 patients could proceed if the predictive probability of success, defined as OR rate (ORR) ≥6% with the lower limit of 95% confidence interval (CI) exceeding 2%, was >80%, assuming a non-informative prior beta (1, 1). With these criteria, there would be 82.0% predictive probability of success if meeting the criterion of 3 of the 40 MSS-CRC patients experiencing an OR at this interim analysis
- PDAC cohort: enrollment of up to 100 patients could proceed if the predictive probability of success, defined as ORR ≥14% with the lower limit of 95% CI exceeding 7.7%, was >80% assuming a non-informative prior beta (1, 1). With these criteria, there would be 85.6% predictive probability of success if meeting the criterion of 7 of the 40 PDAC patients experiencing an OR at this interim analysis
- NSCLC cohort: enrollment of up to 80 patients could proceed if the predictive probability of success, defined as ORR ≥17.5% with the lower limit of 95% CI exceeding 9.8%, was >80% assuming a non-informative prior beta (1, 1). With these criteria, there would be approximately 80% predictive probability of success if meeting the criterion of 8 of the 40 EGFRm NSCLC subjects experiencing an OR at this interim analysis

These criteria were not met at the second interim analysis, and further enrollment to all three cohorts was stopped.

**Analysis populations**

All analyses used the as-treated population, defined as all patients who received ≥1 dose of oleclumab or durvalumab, unless otherwise specified. The DLT-evaluable population included all patients in the escalation phase who received oleclumab or durvalumab per protocol during cycle 1 (two doses) and completed safety follow-up through the DLT-evaluation period from the first dose of oleclumab until the planned administration of the third dose (28 days after first dose or 14 days after second dose), or who experienced any DLT. The response-evaluable population included all patients who had ≥1 post-baseline tumor assessment, who died from any cause, or who discontinued due to clinical disease progression prior to post-baseline tumor assessment.

**Supplementary reference**

1. Rebelatto MC, Midha A, Mistry A et al. (2016) Development of a programmed cell death ligand-1 immunohistochemical assay validated for analysis of non-small cell lung cancer and head and neck squamous cell carcinoma. Diagn Pathol. 11: 95. doi: 10.1186/s13000-016-0545-8

**Supplementary Table 1** Treatment-emergent adverse events

|  | Dose-escalation phase  (*n* = 66) | |  | Expansion phase: oleclumab 40 mg/kg + durvalumab 10 mg/kg  (*n* = 126) | | |
| --- | --- | --- | --- | --- | --- | --- |
| TEAE, *n* (%) | Single-agent oleclumab 5–40 mg/kg  (*n* = 42) | Oleclumab 5–40 mg/kg + durvalumab 10 mg/kg  (*n* = 24) |  | MSS-CRC  (*n* = 42) | PDAC  (*n* = 42) | EGFRm NSCLC  (*n* = 42) |
| Any TEAE | 40 (95) | 24 (100) |  | 40 (95) | 42 (100) | 38 (90) |
| Any Grade 3–4 TEAE | 21 (50) | 18 (75) |  | 22 (52) | 29 (69) | 17 (40) |
| Death due to TEAE | 2 (5) | 0 |  | 1 (2) | 0 | 0 |
| Any serious TEAE | 15 (36) | 10 (42) |  | 17 (40) | 27 (64) | 14 (33) |
| Any TEAE leading to discontinuation^b^ | 2 (5) | 2 (8) |  | 3 (7) | 1 (2) | 2 (5) |
| TEAEs occurring in >10% of patients in any cohort^c^ |  |  |  |  |  |  |
| Fatigue | 17 (40) | 7 (29) |  | 14 (33) | 11 (26) | 12 (29) |
| Vomiting | 8 (19) | 7 (29) |  | 7 (17) | 12 (29) | 3 (7) |
| Abdominal pain | 10 (24) | 5 (21) |  | 10 (24) | 9 (21) | 1 (2) |
| Nausea | 6 (14) | 4 (17) |  | 6 (14) | 12 (29) | 3 (7) |
| Pyrexia | 7 (17) | 3 (13) |  | 6 (14) | 7 (17) | 6 (14) |
| Decreased appetite | 10 (24) | 2 (8) |  | 4 (10) | 9 (21) | 3 (7) |
| Dyspnea | 9 (21) | 3 (13) |  | 6 (14) | 4 (10) | 6 (14) |
| Constipation | 2 (5) | 1 (4) |  | 5 (12) | 9 (21) | 8 (19) |
| AST increased | 2 (5) | 3 (13) |  | 8 (19) | 8 (19) | 3 (7) |
| Anemia | 11 (26) | 2 (8) |  | 6 (14) | 2 (5) | 2 (5) |
| Diarrhea | 3 (7) | 2 (8) |  | 6 (14) | 9 (21) | 3 (7) |
| Blood ALP increased | 3 (7) | 3 (13) |  | 7 (17) | 7 (17) | 0 |
| ALT increased | 4 (10) | 3 (13) |  | 3 (7) | 8 (19) | 0 |
| Back pain | 5 (12) | 1 (4) |  | 4 (10) | 3 (7) | 5 (12) |
| Blood bilirubin increased | 2 (5) | 3 (13) |  | 6 (14) | 4 (10) | 0 |
| Peripheral edema | 4 (10) | 2 (8) |  | 5 (12) | 6 (14) | 1 (2) |
| Cough | 5 (12) | 1 (4) |  | 3 (7) | 4 (10) | 4 (10) |
| GGT increased | 2 (5) | 3 (13) |  | 5 (12) | 7 (17) | 0 |
| Rash | 1 (2) | 2 (8) |  | 3 (7) | 2 (5) | 5 (12) |
| Ascites | 6 (14) | 0 |  | 1 (2) | 5 (12) | 0 |
| Hyponatremia | 3 (7) | 0 |  | 5 (12) | 3 (7) | 1 (2) |
| Pleural effusion | 1 (2) | 0 |  | 3 (7) | 1 (2) | 7 (17) |
| Dizziness | 4 (10) | 0 |  | 1 (2) | 5 (12) | 0 |
| Pulmonary embolism | 1 (2) | 2 (8) |  | 0 | 5 (12) | 2 (5) |
| Depression | 1 (2) | 3 (13) |  | 1 (2) | 1 (2) | 0 |
| Grade 3-4 TEAEs occurring in ≥5% of patients in any cohort^c^ |  |  |  |  |  |  |
| AST increased | 0 | 2 (8) |  | 2 (5) | 4 (10) | 2 (5) |
| Blood ALP increased | 1 (2) | 1 (4) |  | 3 (7) | 5 (12) | 0 |
| Ascites | 5 (12) | 0 |  | 0 | 4 (10) | 0 |
| GGT increased | 1 (2) | 1 (4) |  | 2 (5) | 4 (10) | 0 |
| Pulmonary embolism | 1 (2) | 2 (8) |  | 0 | 4 (10) | 1 (2) |
| Anemia | 2 (5) | 1 (4) |  | 2 (5) | 0 | 2 (5) |
| Blood bilirubin increased | 0 | 2 (8) |  | 3 (7) | 2 (5) | 0 |
| Hyperglycemia | 3 (7) | 1 (4) |  | 1 (2) | 2 (5) | 0 |
| Hyponatremia | 2 (5) | 0 |  | 3 (7) | 1 (2) | 1 (2) |
| Pleural effusion | 1 (2) | 0 |  | 1 (2) | 1 (2) | 4 (10) |
| Abdominal pain | 1 (2) | 1 (4) |  | 0 | 4 (10) | 0 |
| ALT increased | 0 | 2 (8) |  | 2 (5) | 2 (5) | 0 |
| Pneumonia | 0 | 1 (4) |  | 2 (5) | 0 | 3 (7) |
| Hypertension | 1 (2) | 1 (4) |  | 0 | 2 (5) | 1 (2) |
| Sepsis | 1 (2) | 1 (4) |  | 2 (5) | 0 | 1 (2) |
| Vomiting | 0 | 1 (4) |  | 0 | 4 (10) | 0 |
| Lipase increased | 1 (2) | 0 |  | 2 (5) | 0 | 1 (2) |
| Acute kidney injury | 2 (5) | 0 |  | 0 | 0 | 1 (2) |
| Amylase increased | 1 (2) | 0 |  | 0 | 0 | 2 (5) |
| Dyspnea | 1 (2) | 0 |  | 2 (5) | 0 | 0 |
| Small intestinal obstruction | 1 (2) | 0 |  | 2 (5) | 0 | 0 |
| Diarrhea | 0 | 0 |  | 0 | 2 (5) | 0 |
| Hypotension | 2 (5) | 0 |  | 0 | 0 | 0 |
| Pericardial effusion | 0 | 0 |  | 0 | 0 | 2 (5) |
| Serious TEAEs occurring in >1 patient in any cohort^c^  Ascites  Pulmonary embolism  Abdominal pain  Pleural effusion  Pneumonia  Vomiting  Sepsis  Biliary obstruction  Malignant pleural effusion  Small intestinal obstruction  Biliary sepsis  Cholecystitis  Pericardial effusion | 3 (7)  1 (2)  1 (2)  1 (2)  0  0  1 (2)  0  0  1 (2)  0  0  0 | 0  2 (8)  1 (4)  0  1 (4)  1 (4)  1 (4)  1 (4)  1 (4)  0  0  0  0 |  | 1 (2)  0  1 (2)  1 (2)  2 (5)  1 (2)  2 (5)  0  0  2 (5)  0  0  0 | 4 (10)  3 (7)  3 (7)  1 (2)  0  4 (10)  0  2 (5)  0  0  2 (5)  2 (5)  0 | 0  1 (2)  0  3 (7)  3 (7)  0  1 (2)  0  2 (5)  0  0  0  2 (5) |

*AE* adverse event, *ALP* alkaline phosphatase, *ALT* alanine aminotransferase, *AST* aspartate aminotransferase, *CRC* colorectal cancer, *EGFRm* epidermal growth factor receptor-mutant, *GGT* gamma-glutamyl transferase, *MSS-CRC* microsatellite-stable colorectal cancer, *NSCLC* non-small-cell lung cancer, *PDAC* pancreatic ductal adenocarcinoma, *TEAE* treatment-emergent adverse event.

^a^Considered at least possibly related to oleclumab.

^b^Discontinuation of oleclumab. These AEs included small intestinal obstruction and pulmonary embolism (each *n* = 1) in the oleclumab 20 mg/kg monotherapy dose-escalation group; AST increased and blood bilirubin increased in 1 patient in the oleclumab 5 mg combination therapy dose-escalation group, ALT increased and blood alkaline phosphatase increased in 1 patient in the oleclumab 40 mg combination therapy dose-escalation group; eosinophilic fasciitis, systemic inflammatory response syndrome, and peripheral edema in 1 patient each in the CRC expansion cohort, immune-related hepatitis in 1 patient in the PDAC expansion cohort, and hepatitis and renal failure (each *n* = 1) in the NSCLC expansion cohort.

^c^TEAEs and serious TEAEs listed in order of total overall frequency across all cohorts.

**Supplementary Table 2** Treatment-emergent adverse events of special interest for oleclumab and adverse events of special or possible interest for durvalumab. In the dose-escalation phase, none of these adverse events of special interest for oleclumab required systemic steroids or other immunosuppressive treatment. Systemic steroid treatment was required to manage AEs of special or possible interest for durvalumab in 17% of patients receiving combination therapy. No other immunosuppressive medications were required for AEs of special or possible interest for durvalumab in the escalation cohorts. In the expansion phase, systemic steroid treatment was required in 2% of patients to treat AEs of special interest for oleclumab. No AEs of special interest for oleclumab required other immunosuppressive therapy. AEs of special or possible interest for durvalumab required systemic steroid treatment in 10% and other immunosuppressive treatment in 1% of patients in the expansion phase.

|  | Dose-escalation phase  (*n* = 66) | |  | Expansion phase: oleclumab 40 mg/kg + durvalumab 10 mg/kg  (*n* = 126) | | |
| --- | --- | --- | --- | --- | --- | --- |
| AEs, *n* (%) | Single-agent oleclumab 5–40 mg/kg  (*n* = 42) | Oleclumab 5–40 mg/kg + durvalumab 10 mg/kg  (*n* = 24) |  | MSS-CRC  (*n* = 42) | PDAC  (*n* = 42) | EGFRm NSCLC  (*n* = 42) |
| AEs of special interest for oleclumab |  |  |  |  |  |  |
| Any AE of special interest^a^ | 8 (19) | 5 (21) |  | 12 (29) | 16 (38) | 12 (29) |
| Edema peripheral  Pleural effusion  Pulmonary embolism  Deep vein thrombosis  Peripheral swelling  Edema  Embolism  Infusion-related hypersensitivity  Localized edema  Pericardial effusion  Brain edema  Cerebrovascular accident  Cytokine release syndrome  Embolic stroke  Fluid retention  Infusion-related urticaria  Swelling | 4 (10)  1 (2)  1 (2)  0  1 (2)  1 (2)  0  0  0  0  0  0  0  0  1 (2)  0  0 | 2 (8)  0  2 (8)  1 (4)  1 (4)  0  1 (4)  0  0  0  0  0  0  0  0  0  0 |  | 5 (12)  3 (7)  0  0  0  1 (2)  0  2 (5)  2 (5)  0  0  0  1 (2)  0  0  1 (2)  0 | 6 (14)  1 (2)  5 (12)  3 (7)  1 (2)  1 (2)  1 (2)  0  0  0  0  1 (2)  0  1 (2)  0  0  1 (2) | 1 (2)  7 (17)  2 (5)  2 (5)  1 (2)  0  0  0  0  2 (5)  1 (2)  0  0  0  0  0  0 |
| AEs of special or possible interest for durvalumab | | | | | | |
| Any AE of special or possible interest^a^ | NA | 13 (54) |  | 23 (55) | 24 (57) | 16 (38) |
| AST increased  Diarrhea  ALT increased  Blood bilirubin increased  Rash  Pruritus  Arthralgia  Hypothyroidism  Blood creatinine increased  Adrenal insufficiency  Arthritis  Dermatitis acneiform  Hyperbilirubinemia  Hyperthyroidism  Lipase increased  Rash maculo-papular  Amylase increased  Colitis  Hepatic function abnormal  Infusion-related hypersensitivity  Blood TSH increased  Dermatitis  Erythema  Hepatitis  Immune-mediated hepatitis  Infusion-related drug eruption  Infusion-related urticaria  Pancreatitis  Pneumonitis  Rash macular  Rash papular  Systemic inflammatory response syndrome | NA  NA  NA  NA  NA  NA  NA  NA  NA  NA  NA  NA  NA  NA  NA  NA  NA  NA  NA  NA  NA  NA  NA  NA  NA  NA  NA  NA  NA  NA  NA  NA | 3 (13)  2 (8)  3 (13)  3 (13)  2 (8)  2 (8)  2 (8)  0  1 (4)  1 (4)  1 (4)  0  1 (4)  0  0  0  0  0  1 (4)  0  0  0  0  0  0  0  0  0  1 (4)  0  0  0 |  | 8 (19)  6 (14)  3 (7)  6 (14)  3 (7)  3 (7)  1 (2)  2 (5)  0  0  1 (2)  1 (2)  2 (5)  2 (5)  2 (5)  2 (5)  0  0  1 (2)  2 (5)  0  0  1 (2)  0  0  1 (2)  1 (2)  0  0  0  0  1 (2) | 8 (19)  9 (21)  8 (19)  4 (10)  2 (5)  3 (7)  1 (2)  2 (5)  3 (7)  1 (2)  0  1 (2)  0  1 (2)  0  0  0  2 (5)  0  0  1 (2)  0  0  0  1 (2)  0  0  1 (2)  0  1 (2)  1 (2)  0 | 3 (7)  3 (7)  0  0  5 (12)  2 (5)  4 (10)  2 (5)  1 (2)  1 (2)  1 (2)  1 (2)  0  0  1 (2)  1 (2)  2 (5)  0  0  0  0  1 (2)  0  1 (2)  0  0  0  0  0  0  0  0 |

*AE* adverse event, *ALT* alanine aminotransferase, *AST* aspartate aminotransferase, *EGFRm* epidermal growth factor receptor-mutant, *MSS-CRC* microsatellite-stable colorectal cancer, *NA* not applicable, *NSCLC* non-small-cell lung cancer, *PDAC* pancreatic ductal adenocarcinoma.

^a^AEs of special interest for oleclumab or special/possible interest for durvalumab listed in order of total overall frequency across all cohorts.

**Supplementary Table 3** PK parameters for oleclumab in the monotherapy and combination therapy dose-escalation cohorts

|  | Oleclumab monotherapy | | | |  |  | Oleclumab + durvalumab combination therapy | | | |
| --- | --- | --- | --- | --- | --- | --- | --- | --- | --- | --- |
| PK parameter^a^ | Oleclumab  5 mg/kg  (*n* = 3) | Oleclumab  10 mg/kg  (*n* = 11) | Oleclumab  20 mg/kg  (*n* = 12) | Oleclumab  40 mg/kg  (*n* = 16) |  | Oleclumab  5 mg/kg + durvalumab 10 mg/kg  (*n* = 7) | | Oleclumab  10 mg/kg + durvalumab 10 mg/kg  (*n* = 3) | Oleclumab  20 mg/kg + durvalumab 10 mg/kg  (*n* = 4) | Oleclumab  40 mg/kg + durvalumab 10 mg/kg  (*n* = 10) |
| C_max,_ *n*  Geometric mean (µg/mL) (Geometric CV%) | 3 115.2 (27.46) | 11 240.5 (20.80) | 12 410.1 (26.74) | 16 761.8 (26.28) |  | 5 108.7 (12.16) | | 3 195.1 (7.545) | 4 343.7 (39.55) | 9 799.2 (52.15) |
| AUC_(0-14),_ *n* Geometric mean (day*µg/mL)  (Geometric CV%) | 3 413.4 (32.66) | 11 1084 (27.79) | 12 1933 (31.48) | 16 3976 (28.19) |  | 5 428.1 (39.35) | | 3 938.3 (20.97) | 4 2007 (34.60) | 9 3350 (57.04) |
| C_trough,_ *n* Geometric mean (µg/mL)  (Geometric CV%) | 1 NC (NC) | 9 28.29 (69.19) | 10 58.20 (57.12) | 15 128.5 (82.96) |  | 3 5.820 (65.15) | | 3 19.69 (26.33) | 3 61.52 (40.59) | 6 85.65 (90.93) |

*AUC_(0-14)_* area under the concentration time curve from 0 to 14 days post-first dose, *C_max_* maximum observed concentration post-first dose, *C_trough_* lowest observed concentration reached before the second dose is administered, *CV%* geometric coefficient of variation, *NC* not calculated, *PK* pharmacokinetic.

^a^Number of patients with valid observations; three observations were required as a minimum for PK parameters to be summarized.

**Supplementary Fig. 1** Study design: escalation and expansion phases. Overall, a total of 286 patients were screened for the dose-escalation and expansion cohorts, and 94 failed screening due to not meeting the eligibility criteria (*n* = 73), withdrawal of consent (*n* = 8), and other reasons (*n* = 13).
*CRC* colorectal cancer, *DLT* dose-limiting toxicity, *EGFRm* epidermal growth factor receptor-mutant, *IV* intravenous, *MSS* microsatellite-stable, *NSCLC* non-small-cell lung cancer, *PDAC* pancreatic ductal adenocarcinoma, *Q2W* every 2 weeks


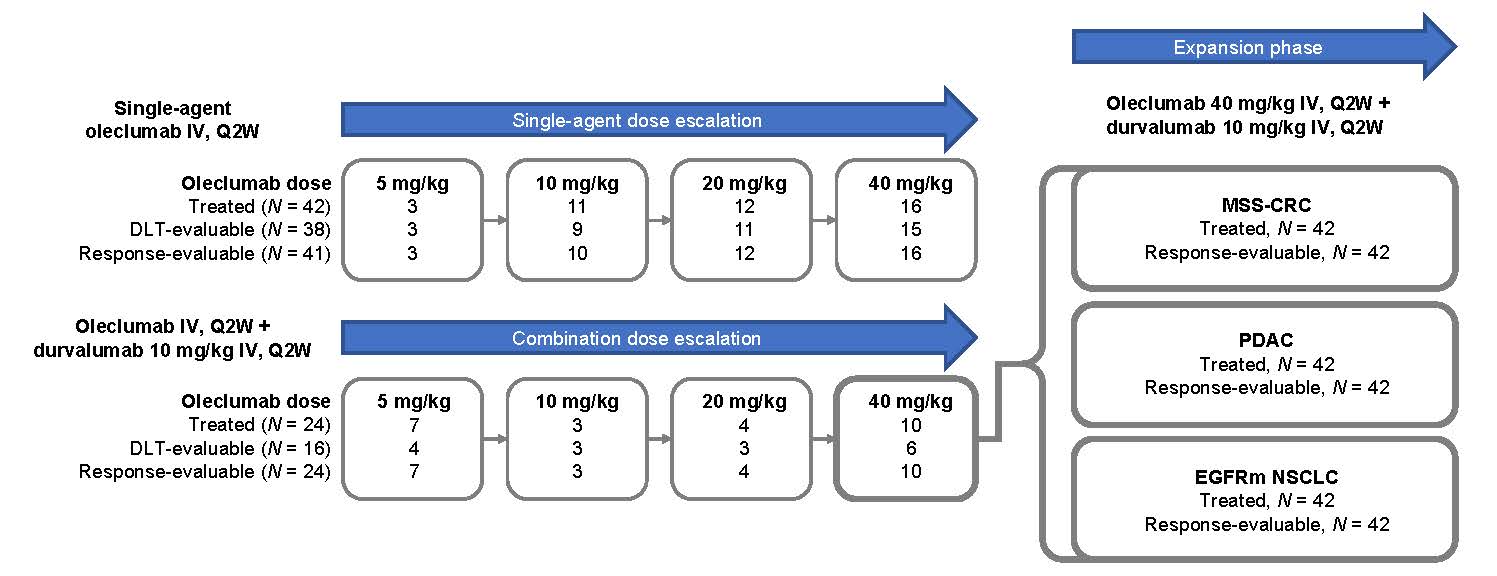


**Supplementary Fig. 2** Oleclumab serum concentrations over time in patients receiving 5–40 mg/kg oleclumab as monotherapy or in combination with durvalumab 10 mg/kg during cycle 1 of the dose-escalation phase (semi-logarithmic scale)


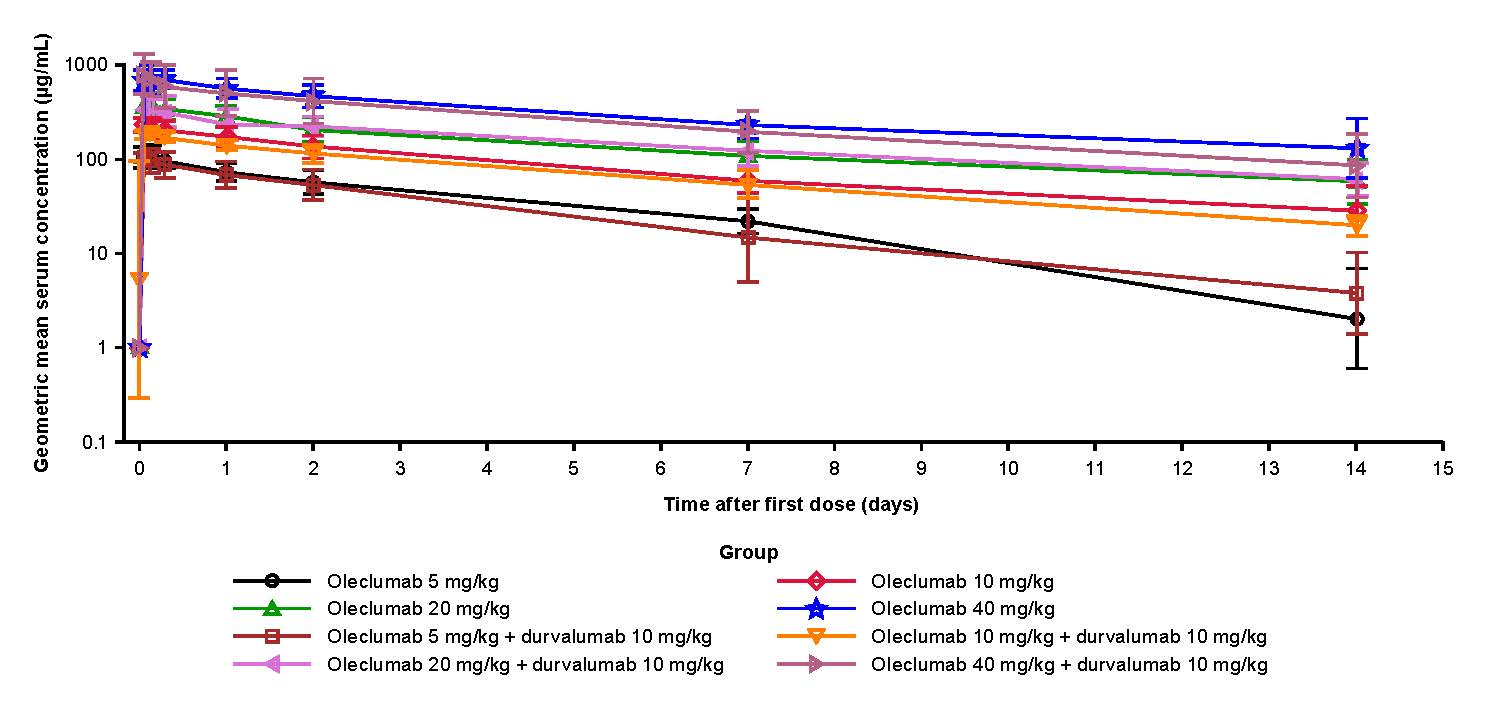
Data shown are geometric mean ± geometric standard deviation.

**Supplementary Fig. 3** Best percentage change in target lesions from baseline in the **a** oleclumab monotherapy and **b** oleclumab plus durvalumab 10 mg/kg dose-escalation cohorts. The patient in the oleclumab 40 mg/kg combination therapy group with a best change from baseline of a >30% reduction in target lesion size (which occurred on day 28, their only post-baseline assessment) was not evaluable for response in non-target lesions and was therefore classified as not evaluable per RECIST.

*CR* complete response, *NE* not evaluable, *PD* progressive disease, *PR* partial response, *SD* stable disease


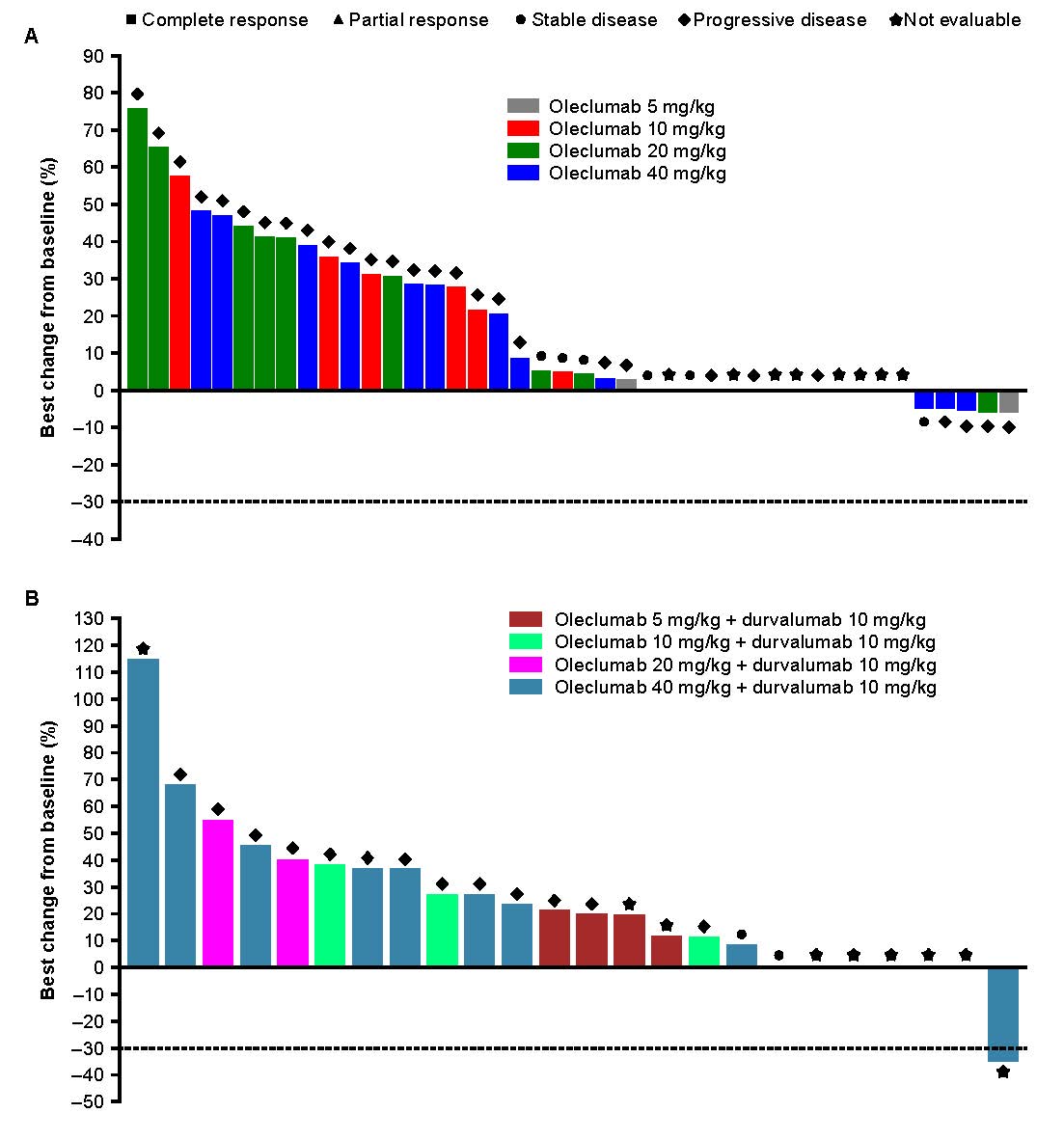


**Supplementary Fig. 4** The relationship between baseline PD-L1 tumor cell expression and best overall response (percent change from baseline in target lesions) in patients with EGFRm NSCLC (dose-expansion cohort).

*PD-L1* programmed cell death ligand-1.


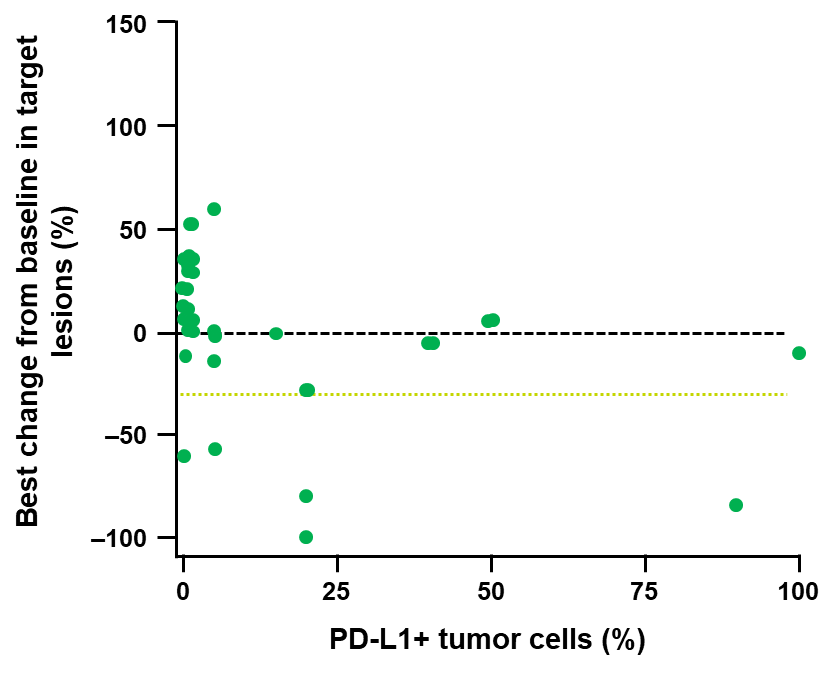

Supplement: Supplementary file 1 — Supplementary file1 (DOCX 372 kb) [file 262_2023_3430_MOESM1_ESM.docx]
